# Deciphering the Genetics of Major End-Use Quality Traits in Wheat

**DOI:** 10.1534/g3.119.400050

**Published:** 2019-02-25

**Authors:** Sepehr Mohajeri Naraghi, Senay Simsek, Ajay Kumar, S.M. Hisam Al Rabbi, Mohammed S. Alamri, Elias M. Elias, Mohamed Mergoum

**Affiliations:** * Department of Plant Sciences, North Dakota State University, Fargo, ND 58108; † Department of Food Sciences and Nutrition, King Saud University, Riyadh 11451, Saudi Arabia; ‡ Department of Crop and Soil Sciences, University of Georgia, Griffin, GA 30223-1797

**Keywords:** genetics, wheat, end-use quality traits, high-density linkage map, quantitative trait loci (QTL), iIdentification, QTL mapping

## Abstract

Improving the end-use quality traits is one of the primary objectives in wheat breeding programs. In the current study, a population of 127 recombinant inbred lines (RILs) derived from a cross between Glenn (PI-639273) and Traverse (PI-642780) was developed and used to identify quantitative trait loci (QTL) for 16 end-use quality traits in wheat. The phenotyping of these 16 traits was performed in nine environments in North Dakota, USA. The genotyping for the RIL population was conducted using the wheat Illumina iSelect 90K SNP assay. A high-density genetic linkage map consisting of 7,963 SNP markers identified a total of 76 additive QTL (A-QTL) and 73 digenic epistatic QTL (DE-QTL) associated with these traits. Overall, 12 stable major A-QTL and three stable DE-QTL were identified for these traits, suggesting that both A-QTL and DE-QTL played an important role in controlling end-use quality traits in wheat. The most significant A-QTL (*AQ.MMLPT.ndsu.1B*) was detected on chromosome 1B for mixograph middle line peak time. The *AQ.MMLPT.ndsu.1B* A-QTL was located very close to the position of the Glu-B1 gene encoding for a subunit of high molecular weight glutenin and explained up to 24.43% of phenotypic variation for mixograph MID line peak time. A total of 23 co-localized QTL loci were detected, suggesting the possibility of the simultaneous improvement of the end-use quality traits through selection procedures in wheat breeding programs. Overall, the information provided in this study could be used in marker-assisted selection to increase selection efficiency and to improve the end-use quality in wheat.

Wheat (*Triticum aestivum* L.) produced in the Northern Great Plains of the USA is known around the world due to its high protein content and outstanding end-use quality traits (Vachal and Benson 2010). In wheat breeding programs, the end-use quality traits are not usually evaluated until late (starting from primarily yield trials onwards) in the breeding program. This is because the end-use quality evaluations are expensive and a large amount of grain is needed to conduct the evaluations. Performing these evaluations at a late stage in the breeding program often results in ostensibly promising wheat lines with high yield and resistance to diseases that cannot be released due to poor end-use quality traits, such as a weak performance for milling parameters and baking properties. To address these challenges, many studies have been conducted to identify quantitative trait loci (QTL) and associated markers for end-use quality traits, with the aim to use such markers in marker-assisted selection (MAS) to improve quality traits in early generations of the breeding program (Campbell *et al.* 2001; Prasad *et al.* 2003; Breseghello *et al.* 2005; Kulwal *et al.* 2005; Arbelbide and Bernardo 2006; Breseghello and Sorrells 2006; Huang *et al.* 2006; Kuchel *et al.* 2006; Kunert *et al.* 2007; Mann *et al.* 2009; Tsilo *et al.* 2010; Zhao *et al.* 2010; Carter *et al.* 2012; Li *et al.* 2012b; Simons *et al.* 2012; El-Feki *et al.* 2015; Deng *et al.* 2015; Echeverry-Solarte *et al.* 2015; Tiwari *et al.* 2016; Jin *et al.* 2016). It should be mentioned that MAS for end-use quality traits would be commenced from F_5_ generation onwards if a single seed decent (SSD) method is used to develop wheat cultivars.

Kernel characteristics, grain protein content; flour, dough, milling, and bread baking characteristics differentiate the end-use quality traits of wheat (*Triticum aestivum* L.) (Souza *et al.* 2002). These traits are complex traits influenced by a combination of environmental conditions and genetic factors (Rousset *et al.* 1992; Peterson *et al.* 1998). Grain protein content has received special attention among end-use quality traits because it is an indication of the quality performance of wheat products such as bread, cake, noodles, and pasta (Zhao *et al.* 2010). Moreover, wheat markets are determined based on the amount of protein in the grain (Regional Quality Report 2011). Several studies reported the existence of genes associated with grain protein content across all wheat chromosomes (Galande *et al.* 2001; Prasad *et al.* 2003; Kulwal *et al.* 2005; Huang *et al.* 2006; Kunert *et al.* 2007; Mann *et al.* 2009; Tsilo *et al.* 2010; Zhao *et al.* 2010; Li *et al.* 2012a and b; Carter *et al.* 2012). Recently, Tiwari *et al.* (2016) reported a major QTL on chromosome 1A associated with grain protein content that account for 16.2–17.7% of the PV across environments using a doubled-haploid population comprised of 138 segregants from a cross between Berkut and Krichauff cultivars. In another study, Boehm *et al.* (2017) identified three major QTL for grain protein content on chromosomes 1A, 7B, and 7B using 132 F6:8 recombinant inbred lines (RILs) population derived from a cross between Butte86 and ND2603. In some of these studies, molecular markers associated with genes regulating gluten proteins have also been reported. Gluten is the coherent mass formed when glutenin and gliadin (storage protein) bind after water is added to flour (Stone and Savin 1999). Glutenins are responsible for dough strength and are composed by subunits of high molecular weight (HMW) and subunits of low molecular weight (LMW). The major genes controlling HMW Glutenins are Glu-1, Glu-A1, Glu-B1, and Glu-D1, whereas the major genes controlling LMW Glutenins are Glu-A3, Glu-B3, and Glu-D3 (Payne 1987).

Mixograph-related properties determine the performance of wheat flour dough during mechanical treatment (Alamri 2009a, b). Mann *et al.* (2009) reported major dough rheology QTL associated with the Glu-B1 and Glu-D1 loci in a double haploid population derived from a cross of Kukri × Jans. The same study also identified a major QTL for unextractable polymeric protein (UPP). Unextractable polymeric proteins were located on chromosomes 1B and 2B and were suggested as a predictor of dough strength (Gras *et al.* 2001). Mann *et al.* (2009) also showed time to peak dough development (TPDD) was associated with the Glu-B1, Glu-B3, and Glu-D1 loci, while peak resistance (PR) was influenced by two QTL detected on chromosome 1A. Several studies have shown the existence of genes associated with flour extraction across all wheat chromosomes except chromosome 1D (Kunert *et al.* 2007; Tsilo *et al.* 2011; Simons *et al.* 2012). Campbell *et al.* (2001) reported several QTL on chromosomes 1B, 3B, 5A, 5B, 5D in a population consisted of 78 F_2:5_ RILs derived from the NY18/CC cross using 370 molecular markers to create a genetic linkage map including restriction fragment length polymorphisms (RFLP), microsatellites, and markers derived from known function genes in wheat. In another study, Echeverry-Solarte *et al.* (2015) identified four stable QTL on chromosomes 1A, 1B, 3D, and 6A for flour extraction in a RIL population derived from a crossing between an elite wheat line (WCB414) and an exotic genotype with supernumerary spikelet. In this study, 939 Diversity Arrays Technology (DArT) markers were used to assemble 38 genetic linkage groups covering 3,114.2 cM with an average distance of 4.6 cM between two markers.

Kuchel *et al.* (2006) identified a major QTL for dough development time on chromosome 1A and several QTL for dough stability time on chromosomes 1A and 1B using two advanced backcross populations named as B22 (Batis × Syn022) and Z86 (Zentos × Syn086). The same study identified QTL for water absorption on chromosomes 1A and 2D (Kuchel *et al.* 2006). Recently, a major QTL for water absorption was detected on the short arm of chromosome 5D using compositions of 390 landraces and 225 released varieties from the wheat germplasm bank of Shandong Academy of Agricultural Science (Li *et al.* 2009). In another study, Li *et al.* (2009) detected a major QTL for water absorption associated with the puroindoline loci on the short arm of chromosome 5D. Further Li *et al.* (2012a) identified a main effect QTL for water absorption on chromosome 5B in two populations derived from crosses among three Chinese wheat cultivars: Weimai8, Jimai20, and Yannong19. Arbelbide and Bernardo (2006) identified four QTL for dough strength on chromosomes 1A, 1B, 1D, and 5B using 80 parental and 373 advanced breeding lines.

Limited information appears to be available on the genetic control of baking properties. Mann *et al.* (2009) found a QTL associated with sponge and dough baking on chromosome 5D in a population of doubled haploid lines derived from a cross between two Australian cultivars Kukri and Janz. In another study, Zanetti *et al.* (2001) detected 10 QTL for dough strength on chromosomes 1B, 5A, 5B, and 5D. Kunert *et al.* (2007) reported two major QTL for loaf volume trait in the BC_2_F_3_ population of B22 (Batis × Syn022). Simons *et al.* (2012) identified a QTL on the long arm of chromosome 1D for bake-mixing time and water absorption traits in a population derived from a cross between BR34 × Grandin. In the same study, Simons *et al.* (2012) found no significant QTL for flour brightness and bake-mixing water absorption, suggesting that these characteristics may be controlled by small effect QTL.

Although several studies were conducted in the past to dissect the genetics of wheat end-use quality traits, almost all of these studies were based on low-density genetic linkage maps containing only several hundred molecular markers. Recently, Boehm *et al.* (2017) conducted a high-density genetic linkage map study that identified 79 QTL associated with end-use quality traits in a wheat RIL population derived from a cross between Butte86 and ND2603 using 607 genotyping-by-sequencing SNP markers, 81 microsatellite markers, and seven HMW and LMW markers. In this study, a total of 35 linkage groups were also assembled with a total map size of 1813.4 cM, an average genetic distance of 2.9 cM between any two markers, and coverage on all wheat chromosomes except chromosome 4D. In another study, Jin *et al.* 2016 performed a high-density linkage map study to detect 119 additive QTL associated with milling quality traits in a RIL population derived from a cross between Gaocheng 8901 and Zhoumai 16. In this study, a total of 46.961 SNP markers based on the wheat Illumina 90K and 660K iSelect SNP assays were used to construct a linkage map with the average density of 0.09 cM per marker.

A low-density genetic linkage map limits the successful application of associated markers in breeding programs. In the current study, the wheat Illumina 90K iSelect assay (Wang *et al.* 2014) was used to detect marker-trait associations for end-use quality traits in wheat. Kumar *et al.* (2016) reported using the wheat Illumina 90K iSelect assay to create a genetic linkage map, indicating that it had a much higher resolution compared to most of the previous genetic linkage maps for the dissection of grain shape and size traits. Thus, the aims of this study were to: (1) construct a high-density linkage map using the wheat Illumina 90K iSelect assay, (2) provide comprehensive insight into the genetic control of end-use quality traits, and (3) identify SNP markers closely linked to QTL associated with end-use quality traits to enhance molecular breeding strategies.

## Material and Methods

### Plant materials

A population of 127 RILs derived from a cross between Glenn (PI-639273; Mergoum *et al.* 2006) and Traverse (PI-642780; resealed by Karl in 2006) was used in this study. Glenn and Traverse are both hard red spring wheat (HRSW) cultivars. Glenn was developed and released in 2005 by the Hard Red Spring Wheat Breeding Program at North Dakota State University (NDSU) in Fargo, ND, USA. It is well-known in domestic and export markets due to its high level of resistance to *Fusarium* head blight (FHB), high grain protein content, and excellent end-use quality characteristics (http://ndsuresearchfoundation.org/glenn). Traverse was developed and released by the South Dakota Agricultural Experiment Station in 2006. It is a high yielding, FHB-tolerant cultivar with marginal grain protein content and end-use quality. The RIL population was advanced by single seed descent (SSD) method from the F2 through F10 generations.

### Field Experiment Design

The RILs, parental lines, and check varieties were grown under field conditions at three locations in ND for three years from 2012 to 2014 (Table 1). In 2012, the three sites were Prosper, Carrington, and Casselton; whereas in 2013 and 2014 the Casselton site was replaced with the Minot site. A detailed description of the environments is given in Table 1. In 2012, lines were grown in a randomized complete block design (RCBD) with two replicates; however, in 2013 and 2014, a 12 × 12 partially balanced square lattice design with two replicates (simple lattice design) was used to reduce experimental error and increase the experiment precision. In 2012 and 2013, each plot was 2.44 m long and 1.22 m wide; whereas in 2014 the plots were 2.44 m long and 1.42 m wide. All plots consisted of seven rows. Sowing rate was 113 kg ha-1 in all environments.

**Table 1:** Description of the environments and planting date to evaluate spring wheat end-use quality traits in a recombinant inbred lines (RIL) population derived from a cross between Glenn and Traverse (NDAWN, 2000-2016)

Location	Year	LAT*^a^*	LNG*^b^*	ALT (m)*^c^*	Planting date	TGS (°C)*^d^*	PGS (mm)*^e^*
Prosper	2012	46°57’46.90”N	97°1’11.31”W	275	05.15.2012	21	148.8
Carrington	2012	47°27’11.56”N	99°9’15.15”W	491	04.23.2012	19	225.0
Casselton	2012	46°51’18.26”N	97°12’39.83”W	283	05.10.2012	21	144.0
Prosper	2013	46°57’46.90”N	97°1’11.31”W	275	05.30.2013	20	318.0
Carrington	2013	47°27’11.56”N	99°9’15.15”W	491	04.30.2013	18	83.2
Minot	2013	48°13’58.68”N	101°17’32.25”W	514	05.14.2013	19	425.0
Prosper	2014	46°57’46.90”N	97°1’11.31”W	275	05.24.2014	19	216.9
Carrington	2014	47°27’11.56”N	99°9’15.15”W	491	05.02.2014	17	203.2
Minot	2014	48°13’58.68”N	101°17’32.25”W	514	05.22.2014	17	347.7

a Latitude in degrees and minutes.

b Longitude in degrees and minutes.

c Altitude in meters.

d Mean temperature during growing season in degrees Celsius (May-October).

e Mean precipitation in growing season in millimeters.

### Phenotypic Data Collection

The grain samples harvested from the field experiments were cleaned in two steps before evaluating quality traits. First, the samples were cleaned using a clipper grain cleaner machine. Second, the samples were cleaned using a carter dockage tester machine. One replicate was used to create a 200-g grain sample per line in each location for evaluating 16 end-use quality characteristics. Quality characteristics analyzed in this study were: grain protein content, flour extraction, eight mixograph-related parameters, and six baking-related properties.

Grain protein content (%) was measured based on 12% moisture using the Near-Infrared Reflectance (NIR) method for protein determination in small grains and following the American Association of Cereal Chemists International (AACCI)-approved method 39-10-01 (AACC International 1999b). Flour extraction (%) was determined using 150 g of thoroughly cleaned wheat grain per sample tempered to 16.0% moisture, using the Brabender Quadrumat Jr. Mill and following the AACCI-approved method 26-50-01 (AACC International 1999d).

Mixograph parameters include the mixograph envelope left slope, mixograph envelope right slope, mixograph MID line peak time, mixograph MID line peak value, mixograph MID line time * value, mixograph MID line peak width, mixograph MID line peak integral, and general mixograph pattern. Mixograph measurements were obtained from 10 g of flour per sample on a 14% moisture basis using the National Manufacturing Mixograph (National Manufacturing, TMCO Division, Lincoln, NE) and following the AACCI-approved method 54-40-02 (AACC International 1999c). Mixsmart software was used to collect data of mixograph envelope left slope (%/min), mixograph envelope right slope (%/min), mixograph MID line peak time (min), mixograph MID line peak value (%), mixograph MID line peak width (%), mixograph MID line peak integral (%/min), and mixograph MID line time * value (%). The general mixograph pattern was based on a 0 to 9 scale (0 = weakest and 9 = strongest) according to USDA/ARS–Western Wheat Quality Laboratory mixogram reference chart (http://wwql.wsu.edu/wp-content/uploads/2017/03/Appendix-6-Mixogram-Chart.pdf).

Baking properties include bake-mixing time, baking absorption, dough character, bread loaf volume, crumb color and crust color, Baking parameters were determined from 100 g of flour per sample on a 14% moisture basis according to the AACCI-approved method 10-09-01 with a little modification in baking ingredients (AACC International 1999a). The baking ingredients were modified as follows: (1) malt dry powder was replaced with fungal amylase (15 SKB); (2) compressed yeast was replaced with instant dry yeast; (3) ammonium phosphate was increased from 0.1 to 5 ppm; (4) two percent shortening was added. Bake mixing time (minutes) was determined as time to full dough development. Baking absorption was evaluated as a percent of flour weight on a 14% moisture basis for the amount of water required for optimum dough baking performance. Dough character was assessed for handling conversion at panning based on a scale of 1 to 10, with higher scores preferred. Bread loaf volume (cubic centimeters) was measured by rapeseed (*Brassica napus* L.) displacement 30 min after the bread was removed from the oven. Crumb color and crust color were valued according to visual comparison with a standard by using a constant illumination source based on a 1 to 10 scale, with higher scores preferred.

### Phenotypic Data Analysis

Because the evaluations of end-use quality are expensive and a large amount of grain is needed, seeds from the two replicates of each environment was bulked and used to analyze phenotypic data. The experimental design employed was a randomized complete block design (RCBD). End-use quality traits analyzed were generated from a bulk sample combining two replicates in each environment, thus data from each environment was considered as a replicate. Variance components were estimated using restricted maximum likelihood (REML) in the MIXED procedure of SAS software Version 9.3 (SAS Institute, Inc., Cary, NC, USA). Blocks (environments) and genotypes were considered random effects. Best linear unbiased predictor (BLUP) values were estimated using the solution option of the random statement of the Proc Mixed procedure in SAS. Broad-sense heritability and genetic correlations were calculated using the Proc Mixed procedure in SAS (Holland *et al.*, 2003; Holland 2006). Broad-sense heritability was estimated as

$$\;H^{2}=\frac{{\hat{\sigma }}_{\text{G}}^{2}}{\left(\frac{{\hat{\sigma }}_{\text{e }}^{2}}{\text{re}}+\frac{{\hat{\sigma }}_{\text{GE}}^{2}}{\text{e}}+{\hat{\sigma }}_{\text{G}}^{2}\right)},$$

where ${\hat{\sigma }}_{\text{G}}^{2}$ is the estimate of genotypic variance, ${\hat{\sigma }}_{\text{GE}}^{2}$ is the estimate of genotype × environment interaction variance, ${\hat{\sigma }}_{\text{e }}^{2}$ is the estimate of error variance, *r* is the number of replications per environment, and *e* is the number of environments. It should be mentioned that, in this study *r* = 1 for the end-use quality traits evaluated on bulked samples. Broad-sense heritability coefficients were classified according to Hallauer and Miranda (1988): VH = very high = H^2^ > 0.70, HI = high = 0.50 < H^2^ < 0.70, M = medium = 0.30 < H^2^ < 0.50, and L = low = H^2^ < 0.30. Pearson correlations between quality traits were evaluated using BLUP values across all environments. The CORR procedure in SAS was used to calculate Pearson correlations. Trait values collected from the first replicate of each environment and BLUP values were used for the QTL mapping analysis.

### Genotyping and Genetic Linkage Map Construction

Lyophilized young leaves were used to isolate genomic DNA for RILs and parental lines following a modified Doyle and Doyle (1987) protocol described by Diversity Arrays Technology Pty., Ltd. (https://ordering.diversityarrays.com/files/DArT_DNA_isolation.pdf). DNA quality was checked via visual observation on 0.8% agarose gel. DNA concentrations were determined with a NanoDrop 1000 spectrophotometer (NanoDrop Technologies, Inc., Wilmington, DE, USA). DNA samples were diluted to the concentration of 50 ng/μl, and 20 μl of the diluted samples were sent to the USDA Small Grains Genotyping Lab in Fargo, ND, for SNP analysis using the wheat Illumina 90K iSelect SNP assay (Wang *et al.* 2014). SNP markers were called as described by Wang *et al.* (2014) using Genome Studio Polyploid Clustering Module v1.0 software (www.illumina.com).

Out of a total 81,587 SNP markers from the wheat Illumina 90K iSelect assay (Wang *et al.* 2014), 8,553 polymorphic SNP markers between parents after excluding poor quality markers were identified. Markers with a high number of missing values (≥ 15%), inconsistent results in three replicates of each parental genotype, or significant segregation distortion (χ2 goodness-of-fit statistic, p < 0.001) were excluded from the following map construction. Linkage analysis for 8,553 SNP markers was performed using a combination of MAPMARKER/EXP software version 3.0 (Lander *et al.* 1987) and MSTmap software (Wu *et al.*, 2008). In the first step, a high-density SNP consensus map was used (Wang *et al.*, 2014) as a reference to select 210 anchor SNP markers for all 21 wheat chromosomes. For each chromosome, 10 SNP markers that covered the whole length of each chromosome were selected. By using MAPMARKER/EXP software version 3.0 (Lander *et al.* 1987) and the 210 anchor SNP markers, 7,963 out of 8,553 SNP markers were placed into the 21 wheat chromosomes based on a minimum LOD score of 5.0 and a maximum distance of 40 centimorgans (cM). In the second step, the marker orders and genetic distances of each linkage group were estimated using MSTmap software (Wu *et al.* 2008), with a cut-off at *P* < 0.000001, the maximum distance of 15 cM between markers, grouping LOD criteria of 5.0, and a minimum linkage group size of 2 cM. Genetic distances between markers were calculated using Kosambi’s genetic mapping function (Kosambi 1944). To check the accuracy of the marker orders, the genetic linkage groups were compared by inspection with the high-density SNP consensus map of Wang *et al.* (2014). The final genetic linkage maps and corresponding graphs were drawn using Mapchart software version 2.2 program (Voorrips 2002).

### Quantitative Trait Loci Mapping

Inclusive composite interval mapping with additive effects (ICIM-ADD) was implemented to identify additive QTL (A-QTL) for each trait within each of the nine environments, as well as across all environments, using QTL IciMapping software version 4.1 (Wang *et al.* 2012). In QTL IciMapping, stepwise regression (p < 0.001) with simultaneous consideration of all marker information was used. The step size chosen for all A-QTL was kept at the default value, 1.0 cM. Left and right confidence intervals were calculated by one-LOD drop off from the estimated A-QTL (Wang *et al.* 2016). The LOD threshold values to detect significant A-QTL were calculated by performing a permutation test with a set of 1,000 iterations at a Type I error of 0.05; all A-QTL identified above the LOD threshold value were reported in this study. In addition, those A-QTL detected in more than two environments or associated with at least two traits were reported. Furthermore, an A-QTL with an average LOD value above the LOD threshold value and an average phenotypic variation (PV) contribution over 10% was considered a major A-QTL. Moreover, A-QTL which were identified in at least three environments were defined as stable QTL.

Inclusive composite interval mapping of epistatic QTL (ICIM-EPI) method, available in QTL IciMapping software version 4.1 (Wang *et al.* 2012), was employed to identify additive-by-additive epistatic interactions or digenic epistatic QTL (DE-QTL) for each of the end-use quality characteristics within each environment, as well as across all environments. For the convenience of illustration, the digenic epistatic QTL were named as DE-QTL. The step size chosen for DE-QTL was 5.0 cM. The probability used in stepwise regression for DE-QTL was 0.0001. To detect DE-QTL, the LOD threshold values were kept at the default value of 5.0. Additionally, the LOD value of 3.0 was also used as another threshold to declare the presence of a putative DE-QTL. Those DE-QTL that were identified in at least two environments were reported in this study. Furthermore, a DE-QTL detected in at least three environments was defined as a stable DE-QTL. It should be noted that in order to represent the most relevant data, only the highest values observed across environments for LOD score, additive effect, epistatic effect, and PV were reported in this study.

### Data Availability

There are two files (Excel files) in the Supplemental Material, File S1 and File S2. File S1 contains three supplementary tables. Supplementary Table 1 includes complete genetic maps developed using Glenn * Traverse RIL population. Supplementary Table 2 shows information related to the complete list of additive QTL (A-QTL) detected for end-use quality traits in a wheat (*Triticum aestivum* L.) RIL population derived from a cross between Glenn (PI-639273) and Traverse (PI-642780). Supplementary Material File S1 shows the complete list of digenic epistatic QTL (DE-QTL) detected for end-use quality traits in a wheat (*Triticum aestivum* L.) RIL population derived from a cross between Glenn and Traverse. File S2 contains genotyping data, linkage groups, and phenotyping data. Supplemental material available at Figshare: https://doi.org/10.25387/g3.6933068.

## Results

### Phenotypic Variation, Heritability, and Genetic and Pearson Correlations

**Figure 1:**
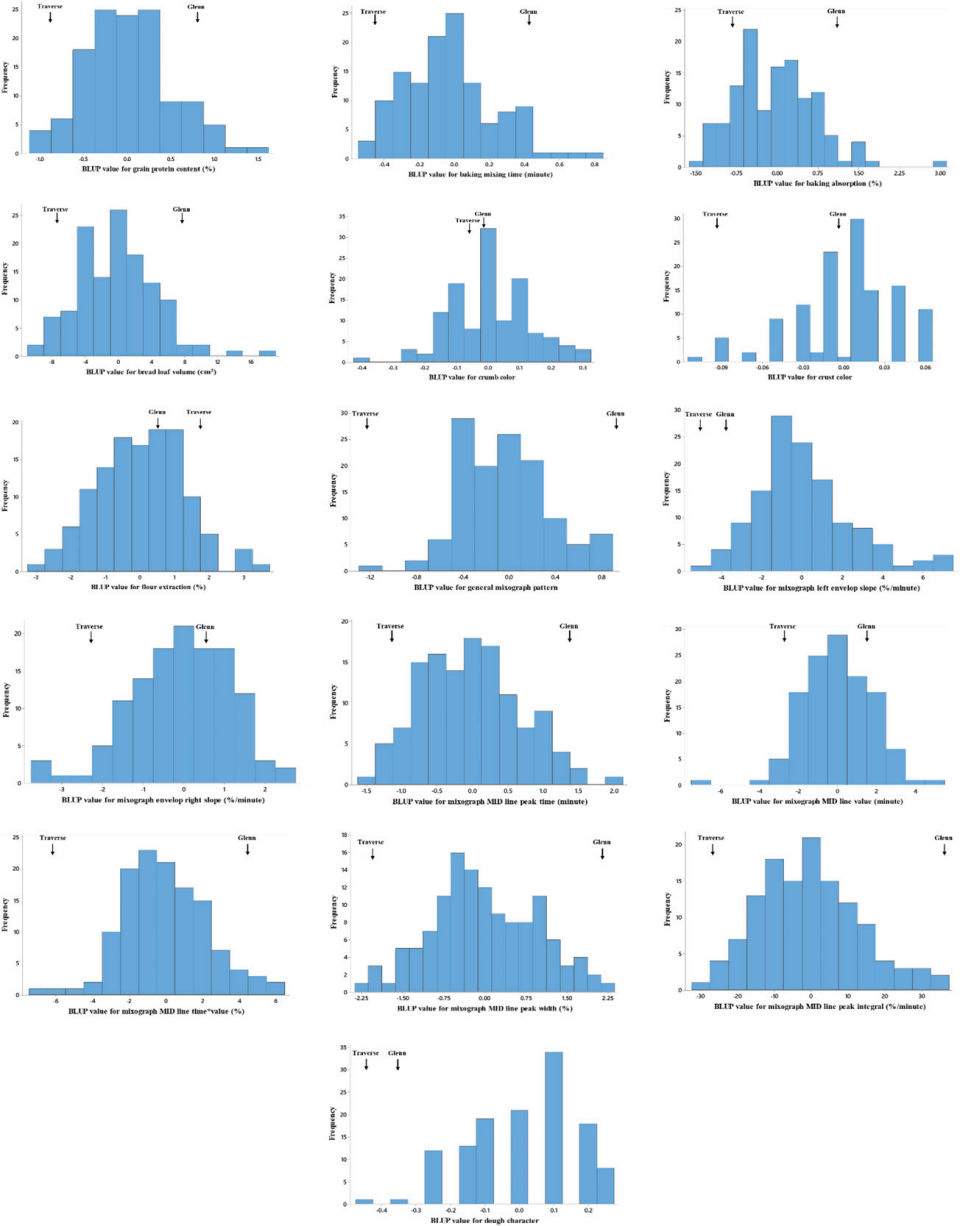
Frequency distribution of BLUP values for end-use quality characteristics of a population of 127 recombinant inbred lines (RILs) derived from a cross between Glenn and Traverse across all environments. Estimates of the parental lines are indicated by arrows.

The RIL population showed variation for all end-use quality characteristics studied (Figure 1; Table 2 and Supplementary Material File S2). The parental lines showed significantly different values for grain protein content, bake-mixing time, baking absorption, bread loaf volume, general mixograph pattern, mixograph envelope left slope, mixograph MID line peak time, mixograph MID line time * value, mixograph MID line peak width, and mixograph MID line peak integral. The values differed slightly but not significantly for crumb color, crust color, flour extraction, mixograph envelope right slope, mixograph MID line peak value, and dough character across all environments (Table 2). All traits showed approximately normal distributions (Figure 1), demonstrating the complex (polygenic) nature and quantitative inheritance of these traits (Fatokun *et al.* 1992). Transgressive segregation in both directions was observed for grain protein content, baking absorption, bread loaf volume, crumb color, flour extraction, mixograph envelope left slope, mixograph envelope right slope, mixograph MID line peak time, and mixograph MID line peak value across all environments, indicating positive alleles were present in both parents. Transgressive segregation for crust color, mixograph MID line time * value, and dough character was observed in the direction of the better parent (Glenn cultivar); several RILs showed better performance than Glenn cultivar for these traits. For flour extraction and mixograph envelope left slope, transgressive segregation in the direction of Traverse was observed, with several RILs showing higher values than the Traverse cultivar for these characteristics (Table 2).

**Table 2:** Phenotypic performance of Glenn, Traverse and their recombinant inbred lines (RILs) based on average / BLUP values and broad-sense heritability (H^2^) for end-use quality traits across all environments

Trait*^a^*	RIL population							
	Glenn	Traverse	Mean	S.D.*^c^*	Range*^d^*	Q_2_*^e^*	H^2^*^f^*	Class of trait H^2^*^g^*
GPC	15.76 /0.51*^b^	14.49 /−0.76	15.25 /0.00	0.50	−1.12 to 1.52	−0.02	0.29	L
BMT	4.08 /0.98*	2.68 /−0.42	3.10 /−0.03	0.26	−0.53 to 0.76	−0.01	0.65	HI
BA	62.44 /1.42*	60.33 /−0.69	61.02 /−0.02	0.75	−1.44 to 2.93	−0.09	0.4	M
BLV	200.83 /6.37*	185.86 /−8.6	194.46 /−0.13	4.67	−10.56 to 17.77	−0.26	0.26	L
CBCL	7.68 /−0.01	7.65 /−0.04	7.69 /0.01	0.12	−0.40 to 0.28	0.02	0.11	L
CTCL	9.63 /−0.01	9.53 /−0.11	9.64 /0.00	0.04	−0.11 to 0.06	0.01	0.05	L
FE	53.51 /0.87	54.07 /1.43	52.64 /−0.01	1.21	−2.91 to 2.89	0.07	0.55	HI
MIXOPA	6.22 /2.93*	2.19 /−1.1	3.29 /−0.04	0.39	−1.19 to 0.82	−0.05	0.42	M
MELS	23.68 /−0.40*	23.70 /−0.38	24.08 /0.19	2.40	−4.64 to 7.18	−0.25	0.38	M
MERS	−10.07 /0.24	−12.44 /−2.13	−10.31 /−0.08	1.21	−3.45 to 2.35	0.01	0.5	HI
MMLPT	5.68 /1.53*	3.10 /−1.05	4.15 /−0.05	0.70	−1.53 to 2.08	−0.09	0.77	VH
MMLPV	60.45 /1.73	55.94 /−2.78	58.72 /0.05	1.85	−6.82 to 5.50	0.16	0.31	M
MMLTV	56.72 /4.23*	45.63 /−6.86	52.49 /−0.06	2.38	−6.52 to 6.48	−0.47	0.41	M
MMLPW	20.79 /2.81*	15.93 /−2.05	17.98 /−0.01	0.96	−2.18 to 2.19	−0.12	0.23	L
MMLPI	185.17 /43.41*	114.29 /−27.47	141.76 /−0.61	13.7	−30.86 to 35.98	−0.77	0.43	M
DO	8.88 /−0.35	8.71 /−0.52	9.23 /0.01	0.16	−0.44 to 0.27	0.01	0.22	L

a GPC: grain protein content, BMT: bake mixing time, BA: baking absorption, BLV: bread loaf volume, CBCL: crumb color, CTCL: crust color, FE: flour extraction rate, MIXOPA: the general mixograph pattern, MELS: mixograph envelope left slope, MERS: mixograph envelope right slope, MMLPT: mixograph MID line peak time, MMLPV: mixograph MID line peak value, MMLTV: mixograph MID line time * value, MMLPW: mixograph MID line peak width, MMLPI: mixograph MID line peak integral; DO: dough character.

b *A significant difference between parental lines at *P* < 0.05.

c Standard deviation.

d Range is estimated based on BLUP values.

e The second quartile or median.

f broad-sense heritability coefficient according to Holland (2003).

g Class of broad-sense heritability according to Hallauer and Miranda (1988), VH = very high = H^2^ > 0.70, HI = high = 0.50 < H^2^ < 0.70, M = medium = 0.30 < H^2^ < 0.50, L = low = H^2^ <0.30.

The broad-sense heritability coefficients varied substantially for different traits. The highest estimated broad-sense heritability was for mixograph MID line peak time (0.77), and the lowest for crust color (0.05) (Table 2). Among baking properties, bake-mixing time and baking absorption showed high and moderate broad-sense heritability (0.65 and 0.40, respectively); while bread loaf volume, crumb color, crust color, and dough character showed low broad-sense heritability (0.26, 0.11, 0.05, and 0.22, respectively). Among milling and mixograph traits, flour extraction, general mixograph pattern, mixograph envelope left slope, mixograph envelope right slope, mixograph MID line peak time, mixograph MID line peak value, mixograph MID line time * value, and mixograph MID line peak integral showed moderate to high broad-sense heritability (0.55, 0.42, 0.38, 0.50, 0.77, 0.31, 0.41, and 0.43, respectively), but mixograph MID line peak width had low broad-sense heritability (0.23). High to very high broad-sense heritability coefficients for bake-mixing time, flour extraction, mixograph MID line peak time, and mixograph MID line peak value indicated stability of these traits, and the PV of these characteristics was mainly due to genetic effects (Table 2).

The genetic and Pearson correlation analyses showed most of the quality traits were associated with each other (Table 3). Highly positive significant genetic and phenotypic correlations (correlation coefficient value lies between + 0.50 and + 0.97) were observed between grain protein content and bread loaf volume; grain protein content and envelope left slope; grain protein content and mixograph MID line peak value; bake-mixing time and general mixograph pattern; bake-mixing time and mixograph envelope right slope; bake-mixing time and mixograph MID line peak time; bake-mixing time and mixograph MID line peak integral; baking absorption and mixograph MID line peak value; bread loaf volume and mixograph envelope left slope; general mixograph pattern and mixograph MID line time * value; general mixograph pattern and mixograph MID line peak width; general mixograph pattern and mixograph MID line peak integral; mixograph envelope right slope and mixograph MID line peak time; mixograph MID line peak time and mixograph MID line peak integral; and mixograph MID line peak integral; mixograph MID line time * value and mixograph MID line peak width; and mixograph MID line time * value and mixograph MID line peak integral. In contrast, high negative significant genetic and phenotypic correlations (correlation coefficient value lies between - 0.50 and – 0.87) were found between bake-mixing time and mixograph envelope left slope; mixograph envelope left slope and mixograph MID line peak time; and mixograph envelope right slope and mixograph MID line peak value. Moderate positive significant genetic and phenotypic correlations, where correlation coefficient value lies between + 0.30 and + 0.50 and is significant at *P* < 0.01, were identified between grain protein content and mixograph MID line time * value; grain protein content and mixograph MID line peak width; bake-mixing time and mixograph MID line time * value; bake-mixing time and mixograph MID line peak width; baking absorption and mixograph envelope left slope; bread loaf volume and crust color; NLV and general mixograph pattern; bread loaf volume and mixograph MID line peak value; crust color and general mixograph pattern; crust color and mixograph MID line peak value; crust color and mixograph MID line time * value; crust color and mixograph MID line peak width ; general mixograph pattern and mixograph MID line peak time; general mixograph pattern and mixograph MID line peak value; mixograph envelope right slope and mixograph MID line peak integral; mixograph MID line peak time and mixograph MID line time * value; and mixograph MID line peak width and mixograph MID line peak integral. However, moderate negative but highly significant genetic and phenotypic correlations (correlation coefficient value lies between - 0.30 and - 0.50) were detected between grain protein content and mixograph envelope right slope; grain protein content and mixograph MID line peak time; bake-mixing time and mixograph envelope left slope; baking absorption and mixograph MID line peak time; mixograph MID line peak time and mixograph MID line peak value. In other pairs of traits genetic and phenotypic correlations were either low or not statistically significant at *P* < 0.05. Correlations between the end-use quality traits are shown in more detail in Table 3. Differences between genetic and phenotypic correlation coefficients (Table 3) could be due to low heritability values; Hill and Thompson (1978) suggested higher heritability values could result in the accuracy of genetic correlation estimates and greater similarity of genetic and phenotypic correlation coefficients. The overall level of genetic correlation was greater than phenotypic correlation, but the magnitude and pattern of genetic and phenotypic correlations were similar, suggesting phenotypic correlations would likely be fair estimates of their genetic correlations in end-use quality traits (Table 3).

**Table 3:** Genetic and Pearson’s rank correlations of end-use quality traits for the recombinant inbred lines (RILs) population derived from a cross between Glenn and Traverse across all environments. Values in bold displayed above the diagonal indicate genetic correlation coefficients, and values under the diagonal show Pearson correlation coefficients

Trait*^a^*	GPC	BMT	BA	BLV	CBCL	CTCL	FE	MIXOPA	MELS	MERS	MMLPT	MMLPV	MMLTV	MMLPW	MMLPI	DO
GPC	—	−0.29**^c^	0.42**	0.76**	**0.18**	0.48**	−0.31**	0.25**	0.70**	−0.49**	−0.35**	0.74**	0.34**	0.40**	**0.11**	**0.10**
BMT	−0.29**^b^	—	**−0.17**	−0.29**	**−0.05**	0.27**	**−0.02**	0.73**	−0.60**	0.69**	0.90**	**−0.11**	0.69**	0.50**	0.89**	0.27**
BA	0.33**	−0.12	**—**	0.22*	0.21*	**0.14**	−0.53**	0.31**	0.61**	−0.36**	−0.46**	0.80**	0.37**	0.32**	**0.01**	**0.07**
BLV	0.59**	−0.24**	0.16	—	0.29**	0.97**	**−0.08**	0.43**	**0.76**	−0.37**	−0.22*	0.70**	0.38**	0.48**	0.24**	**0.01**
CBCL	0.13	−0.06	0.10	0.23**	**—**	0.39**	−0.41**	**0.10**	0.24**	**0.13**	**−0.05**	**0.08**	**0.10**	**−0.03**	**0.13**	−0.30**
CTCL	0.21*	0.06	0.06	0.34**	0.07	—	−0.49**	0.65**	0.48**	**0.10**	**0.16**	0.62**	0.71**	0.71**	0.65**	−0.65**
FE	−0.24**	−0.04	−0.36**	−0.05	−0.20*	−0.16	**—**	−0.20*	**−0.18**	**−0.02**	**0.07**	−0.25**	−0.25**	−0.35**	**−0.16**	**−0.13**
MIXOPA	0.24**	0.57**	0.22*	0.30**	0.07	0.37**	−0.14	—	**−0.13**	0.58**	0.58**	0.45**	0.97**	0.83**	0.92**	**0.08**
MELS	0.57**	−0.48**	0.41**	0.46**	0.14	0.17	−0.11	0.01	**—**	−0.87**	−0.79**	0.79**	**−0.03**	**0.09**	−0.43**	**0.03**
MERS	−0.48**	0.55**	−0.27**	−0.26**	0.01	0.02	−0.03	0.25**	−0.67**	—	0.83**	−0.67**	0.33**	**0.16**	0.61**	**−0.02**
MMLPT	−0.35**	0.85**	−0.39**	−0.19*	−0.06	0.05	0.04	0.44**	−0.64**	0.68**	**—**	−0.48**	0.45**	0.36**	0.97**	0.24**
MMLPV	0.62**	−0.11	0.48**	0.39**	0.01	0.30**	−0.14	0.42**	0.61**	−0.54**	−0.31**	**—**	0.49**	0.83**	**−0.03**	−0.21*
MMLTV	0.33**	0.48**	0.26**	0.24**	0.02	0.33**	−0.17	0.79**	0.08	0.11	0.36**	0.67**	—	0.96**	0.80**	**0.11**
MMLPW	0.35**	0.31**	0.20*	0.27**	0.02	0.35**	−0.19*	0.66**	0.13	−0.04	0.18*	0.60**	0.71**	—	0.71**	**−0.17**
MMLPI	0.04	0.67**	0.03	0.10	0.04	0.14	−0.17	0.62**	−0.29**	0.41**	0.75**	0.01	0.53**	0.34**	—	0.56**
DO	0.13	0.09	0.02	0.03	−0.03	−0.09	−0.04	0.11	0.05	−0.11	0.14	0.05	0.15	0.04	0.24**	—

a GPC: grain protein content, BMT: bake mixing time, BA: baking absorption, BLV: bread loaf volume, CBCL: crumb color, CTCL: crust color, FE: flour extraction rate, MIXOPA: the general mixograph pattern, MELS: mixograph envelope left slope, MERS: mixograph envelope right slope, MMLPT: mixograph MID line peak time, MMLPV: mixograph MID line peak value, MMLTV: mixograph MID line time * value, MMLPW: mixograph MID line peak width, MMLPI: mixograph MID line peak integral; DO: dough character.

b Genetic correlation coefficient according to Holland 2006.

c Pearson correlation based on BLUP values.

* and ** Significant at *P <* 0.05 and 0.01, respectively; ^ns^ not significant at *P <* 0.05.

### Genetic Linkage Map

Out of a total of 8,553 SNP markers, 7,963 markers were selected for genetic linkage mapping according to criteria described in the materials and methods section (Supplementary Material File S2). These markers were mapped onto 41 linkage groups covering all 21 wheat chromosomes (Table 4 and Supplementary Material File S1 and File S2). The linkage maps covered a total genetic length of 2,644.82 cM, with an average distance of 0.33 cM between any two markers (Table 4 and Supplementary Material File S1). The linkage map consisted of 1,427 loci (∼18%), with an average genetic distance of 1.85 cM between any two loci. Altogether, the B-genome contained considerably more markers (4,807) than the A-genome (2,549); notably fewer markers were mapped on the D-genome (607). The number of markers on individual linkage groups varied from 10 (1B2) to 770 (3B1). Furthermore, the number of loci in a linkage group ranged from 2 (3D1) to 113 (7A1) (Table 4). The map position of each chromosome of Glenn/Traverse map was compared with the high-density SNP consensus map of Wang *et al.* (2014). The results showed that the marker orders were fairly consistent with the average Spearman’s rank-order correlation coefficient of 0.83.

**Table 4:** Distribution of markers and marker density across linkage groups in the bread wheat (*Triticum aestivum* L.) genetic map developed by using the recombinant inbred line (RIL) population of a cross between Glenn (PI-639273) and Traverse (PI-642780)

Linkage group	No. of markers	No. of loci	Map distance (cM)	Map density (cM/marker)	Map density (cM/locus)
1A1	345	70	131.08	0.38	1.87
1A2	108	24	30.79	0.29	1.28
2A1	215	74	142.28	0.66	1.92
2A2	52	11	14.30	0.28	1.30
3A1	221	41	87.52	0.40	2.13
3A2	91	18	60.99	0.67	3.39
4A1	278	57	150.56	0.54	2.64
5A1	78	21	80.58	1.03	3.84
5A2	197	42	59.79	0.30	1.42
5A3	29	14	32.84	1.13	2.35
6A1	173	33	72.94	0.42	2.21
6A2	173	23	16.24	0.09	0.71
7A1	525	113	196.80	0.37	1.74
7A2	64	18	17.14	0.27	0.95
1B1	529	58	68.48	0.13	1.18
1B2	10	5	19.69	1.97	3.94
1B3	43	10	11.10	0.26	1.11
2B1	461	54	40.33	0.09	0.75
2B2	614	106	181.12	0.29	1.71
3B1	770	70	77.38	0.10	1.11
3B2	78	21	31.31	0.40	1.49
3B3	27	9	16.27	0.60	1.81
3B4	103	29	18.45	0.18	0.64
4B1	273	58	111.08	0.41	1.92
5B1	395	88	241.74	0.61	2.75
6B1	794	103	144.16	0.18	1.40
6B2	104	22	73.09	0.70	3.32
7B1	555	88	134.67	0.24	1.53
7B2	51	14	11.12	0.22	0.79
1D1	111	24	78.26	0.71	3.26
2D1	131	7	13.48	0.10	1.93
2D2	47	16	14.09	0.30	0.88
2D3	11	10	22.03	2.00	2.20
3D1	33	2	9.62	0.29	4.81
4D1	17	7	6.21	0.37	0.89
5D1	118	12	21.32	0.18	1.78
6D1	40	14	73.50	1.84	5.25
6D2	31	10	10.75	0.35	1.08
7D1	31	14	35.44	1.14	2.53
7D2	22	5	9.89	0.45	1.98
7D3	15	12	76.40	5.09	6.37
A genome	2549	559	1093.86	0.43	1.96
B genome	4807	735	1179.99	0.25	1.61
D genome	607	133	370.97	0.61	2.79
Whole genome	7963	1427	2644.82	0.33	1.85

### Quantitative Trait Loci Analysis

A total of 76 A-QTL and 73 DE-QTL were identified for the 16 end-use quality traits evaluated in this study (Table 5; Table 6 and Supplementary Material File S1). These A-QTL and DE-QTL were distributed across all wheat chromosomes except chromosomes 3D and 6A for A-QTL, and 3D for DE-QTL. In terms of the genome-wide distribution of QTL, the B-genome had the highest number of A-QTL (36), while the A-genome had the most DE-QTL (46). This was followed by the A-genome with 25 A-QTL, the D-genome with 15 A-QTL, the B-genome with 23 DE-, and the D-genome with four DE-QTL (Table 5 and Table 6). All of the A-QTL and DE-QTL were identified in at least two environments and/or were associated with at least two different end-use quality traits (Table 5 and Table 6). Out of the 76 A-QTL, a total of 43 A-QTL (∼57%) explained more than 10% of PV and were considered major A-QTL, while the remaining 32 A-QTL explained less than 10% of PV and were considered minor QTL (Table 5). Furthermore, a total of 12 A-QTL and three DE-QTL were identified in at least three environments and were considered stable QTL.

**Table 5:** QTL detected for end-use quality traits in a bread wheat (*Triticum aestivum* L.) RIL population derived from a cross between Glenn (PI-639273) and Traverse (PI-642780)

Trait*^a^*	A-QTL name	Other associated traits	Env.*^b^*	Chromosome/linkage group	Left marker	Right marker	Position (cM)*^c^*	LOD*^d^*	Additive effect	PV(%)*^e^*	Confidence intervals	Previously identified A-QTL in the same chromosome region
FE	*AQ.FE.ndsu.1A.1*	—	I, X	1A1	BS00084022_51	RAC875_c9700_989	50	8.7788	−0.4935	14.4911	48.5-50.5	—
FE	*AQ.FE.ndsu.1A.2*	GPC	VII	1A1	wsnp_Ra_c15564_23999084	wsnp_BG263358A_Ta_2_3	94	7.6547	−1.061	19.4012	92.5-95.5	—
GPC	*AQ.GPC.ndsu.1A*	FE	III, V, VIII, VIIII	1A1	wsnp_Ra_c15564_23999084	wsnp_BG263358A_Ta_2_3	95	4.6476	0.2376	13.6941	93.5-96.5	—
BMT	*AQ.BMT.ndsu.1B*	MMLPI	IV, VIII, VIIII	1B1	TA015141-0717	wsnp_JD_c4444_5575748	13	4.7945	0.1736	12.9075	12.5-13.5	—
BMT	*AQ.BMT.ndsu.1B.1*	—	VI, X	1B1	Kukri_c33561_564	wsnp_Ku_c16938_25916260	14	13.6184	0.1303	12.085	13.5-14.5	—
BMT	*AQ.BMT.ndsu.1B.2*	MMLPT	I, V	1B1	RAC875_c75885_302	Tdurum_contig28305_106	20	6.5489	0.1804	12.5043	19.5-20.5	—
GPC	*AQ.GPC.ndsu.1B.1*	MIXOPA	VII	1B1	BS00064162_51	Excalibur_rep_c101787_89	57	3.9039	0.2683	8.1766	56.5-58.5	—
MIXOPA	*AQ.MIXOPA.ndsu.1B.1*	GPC	IV	1B1	BS00064162_51	Excalibur_rep_c101787_89	57	3.9039	0.2683	7.7358	56.5-58.5	—
MMLPI	*AQ.MMLPI.ndsu.1B.1*	BMT	VI, VIII, X	1B1	TA015141-0717	wsnp_JD_c4444_5575748	13	7.5203	10.7587	15.9048	12.5-13.5	—
MMLPI	*AQ.MMLPI.ndsu.1B.2*	MMLPT; MMLTV; BMT	IV	1B1	RAC875_c75885_302	Tdurum_contig28305_106	20	14.3296	33.5754	16.6441	19.5-20.5	—
MMLPT	*AQ.MMLPT.ndsu.1B*	BMT	I, IV, V, VI, VII, VIII, VIIII, X	1B1	RAC875_c75885_302	Tdurum_contig28305_106	20	15.2002	0.3698	24.4267	19.5-20.5	Jin *et al.* 2016
MMLPW	*AQ.MMLPW.ndsu.1B*	—	V, X	1B1	wsnp_Ex_c2569_4780450	Tdurum_contig65853_534	62	4.6175	0.3643	11.4578	60.5-65.5	—
MMLTV	*AQ.MMLTV.ndsu.1B*	MMLPI; MMLPT; BMT	IV	1B1	RAC875_c75885_302	Tdurum_contig28305_106	20	4.3062	12.1355	1.6784	19.5-20.5	—
BA	*AQ.BA.ndsu.1B*	—	I, IV, VIII, III	1B3	BS00093275_51	BobWhite_c12960_138	0	3.6756	−0.4042	8.1774	0-2.5	Tsilo *et al.* 2011
BMT	*AQ.BMT.ndsu.1D*	—	VIII, X	1D1	RAC875_rep_c105196_532	BS00038418_51	76	25.0366	0.1984	27.7923	74.5-76.5	—
BMT	*AQ.BMT.ndsu.2A.1*	GPC	I	2A1	Excalibur_c27279_699	Kukri_c44255_832	37	8.2391	−0.204	12.8403	34.5-38.5	—
FE	*AQ.FE.ndsu.2A.1*	MMLPT	V	2A1	BS00022903_51	Ra_c34214_1320	20	7.9438	0.8736	10.3544	19.5-22.5	—
GPC	*AQ.GPC.ndsu.2A.1*	BMT	IV,V	2A1	Kukri_c44255_832	RAC875_c13861_1248	38	6.2687	0.4351	13.19	37.5-39.5	—
GPC	*AQ.GPC.ndsu.2A.2*	MMLPT	III, X	2A1	wsnp_Ex_c28204_37349164	Kukri_c77188_798	18	4.939	0.1596	8.0024	17.5-19.5	—
MMLPT	*AQ.MMLPT.ndsu.2A.1*	GPC	I, III	2A1	wsnp_Ex_c28204_37349164	Kukri_c77188_798	18	5.2543	−0.5361	16.3459	17.5-19.5	—
MMLPT	*AQ.MMLPT.ndsu.2A.2*	FE	III	2A1	BS00022903_51	Ra_c34214_1320	20	7.9438	0.8736	10.0223	19.5-22.5	—
GPC	*AQ.GPC.ndsu.2B*	—	I, III	2B2	BS00064658_51	RAC875_c1755_971	27	4.6386	−0.1599	8.7567	23.5-27.5	—
BLV	*AQ.BLV.ndsu.2D.1*	—	II, X, III	2D2	Kukri_c31121_1460	Kukri_c44769_750	7	3.8365	4.4342	9.7413	5.5-8.5	—
BLV	*AQ.BLV.ndsu.2D.2*	—	VII, VIII	2D2	BobWhite_c6365_965	D_GDS7LZN02FDZX8_269	4	3.6217	8.5516	12.8348	3.5-4.5	Tsilo *et al.* 2011
MMLPT	*AQ.MMLPT.ndsu.2D*	—	II, IV, VII, X	2D3	BS00011109_51	wsnp_Ku_c8712_14751858	20	4.3893	−0.1918	6.5246	13.5-22	—
BMT	*AQ.BMT.ndsu.3A*	MMLPT	I, V, VIIII, X	3A2	BobWhite_c38444_238	Kukri_c10751_1031	47	12.0827	0.1218	10.2537	46.5-48.5	—
GPC	*AQ.GPC.ndsu.3A*	—	III, V, X	3A2	BS00022058_51	Excalibur_c39808_453	26	5.9339	−0.334	9.3796	21.5-28.5	—
MMLPT	*AQ.MMLPT.ndsu.3A.1*	BMT	IV, VIIII, X	3A2	Kukri_c10751_1031	wsnp_Ex_c1533_2930233	49	6.8915	0.2345	9.5047	47.5-51.5	—
GPC	*AQ.GPC.ndsu.3B.1*	MMLPV	X	3B1	wsnp_Ex_c47078_52393295	D_GB5Y7FA01EIDVZ_263	25	7.5082	0.206	13.0023	22.5-27.5	—
MMLPV	*AQ.MMLPV.ndsu.3B.1*	GPC	VIII	3B1	RFL_Contig1456_842	wsnp_Ex_c47078_52393295	24	5.3548	2.4546	7.5943	22.5-27.5	—
FE	*AQ.FE.ndsu.3B*	—	I, V, VII, X	3B1	Tdurum_contig82214_79	wsnp_BE499016B_Ta_2_1	68	8.5226	−0.5046	15.2959	64.5-69.5	Carter *et al.* 2012
BMT	*AQ.BMT.ndsu.3B.1*	—	II, V, X	3B2	Tdurum_contig12455_385	Excalibur_c21708_555	0	4.9225	0.0716	3.5988	0-0.5	—
BMT	*AQ.BMT.ndsu.3B.2*	MMLPI; MMLTV	I, VIII	3B2	Excalibur_rep_c102270_677	Kukri_c2227_583	6	7.7153	0.1939	11.5294	5.5-6.5	—
MMLPI	*AQ.MMLPT.ndsu.3B.2*	BMT; MMLTV;	IV	3B2	Excalibur_rep_c102270_677	Kukri_c2227_583	6	4.9406	8.976	9.6693	5.5-6.5	—
MMLPT	*AQ.MMLPT.ndsu.3B.2*	—	IV, VI, VIII, X	3B2	Tdurum_contig15928_135	BobWhite_c9424_243	5	3.8931	0.1712	5.1946	4.5-5.5	—
MMLTV	*AQ.MMLTV.ndsu.3B.2*	BMT; MMLTV;	V	3B2	Excalibur_rep_c102270_677	Kukri_c2227_583	6	3.4132	2.3331	9.894	5.5-6.5	—
BA	*AQ.BA.ndsu.4A*	FE; MMLTV	IV, VI, X	4A1	BS00022395_51	BS00021957_51	147	6.6931	0.2547	11.552	144.5-148.5	Jin *et al.* 2016
MMLPV	*AQ.MMLPV.ndsu.4A*	—	VII, X	4A1	TA004912-0408	Kukri_c17417_797	150	5.821	0.8158	13.7424	149.5-150	—
MMLTV	*AQ.MMLTV.ndsu.4A*	FE; BA	IV, V, X	4A1	Kukri_c35451_857	BS00022395_51	143	3.5732	0.7363	7.8228	141.5-145.5	—
FE	*AQ.FE.ndsu.4A.1*	MMLTV;BA	X	4A1	Kukri_c18346_556	Kukri_c35451_857	142	4.5021	−0.3776	6.9089	141.5-144.5	—
BLV	*AQ.BLV.ndsu.4B.1*	BMT	VI, X	4B1	RAC875_c39339_400	RAC875_c17026_714	97	4.0885	−1.3594	7.4436	94.5-97.5	—
BMT	*AQ.BMT.ndsu.4B.1*	BLV	III, X	4B1	RAC875_c39339_400	RAC875_c17026_714	97	4.0885	−1.3594	6.7181	94.5-97.5	—
GPC	*AQ.GPC.ndsu.4B1*	—	I, II	4B1	BobWhite_c47144_153	Tdurum_contig10302_187	94	6.6325	−0.2086	15.0008	93.5-94.5	—
BA	*AQ.BA.ndsu.4B.1*	MIXOPA	V	4B1	Excalibur_c39876_403	Kukri_c19909_733	70	4.7301	−0.6243	11.2395	69.5-73.5	—
MIXOPA	*AQ.MIXOPA.ndsu.1B.1*	BA	II	4B1	Excalibur_c39876_403	Kukri_c19909_733	70	5.0876	−0.2838	12.3347	69.5-70.5	—
BA	*AQ.BA.ndsu.4D.1*	MELS; MERS	I, III, V, VIIII, X	4D1	wsnp_JD_rep_c51623_35119179	Ra_c350_837	1	14.2653	−0.3725	28.0617	0-1.5	—
MELS	*AQ.MELS.ndsu.4D.1*	BA; MERS	III, X	4D1	wsnp_JD_rep_c51623_35119179	Ra_c350_837	1	6.6917	−3.0005	18.0403	0-1.5	—
MERS	*AQ.MERS.ndsu.4D.1*	BA; MELS	IV, X	4D1	wsnp_JD_rep_c51623_35119179	Ra_c350_837	1	3.6362	0.4349	13.0994	0-2.5	—
BLV	*AQ.BLV.ndsu.5A*	—	IV,VI	5A1	Kukri_c28555_114	wsnp_Ku_c18023_27232712	36	6.9598	−5.0049	15.8001	30.5-42.5	—
BLV	*AQ.BLV.ndsu.5B*	GPC	X	5B1	BS00064297_51	wsnp_BE499835B_Ta_2_5	25	5.5542	18.5451	2.1438	11.5-35.5	—
FE	*AQ.FE.ndsu.5B*	—	V, X	5B1	Kukri_c3070_72	BS00021993_51	240	3.4037	0.2971	5.1324	238.5-241	—
GPC	*AQ.GPC.ndsu.5B*	BLV	I, II, IV, V, VII, VIIII, X	5B1	BS00032003_51	wsnp_BE499835B_Ta_2_5	14	10.3662	0.3196	20.1838	9.5-20.5	—
MIXOPA	*AQ.MIXOPA.ndsu.5B*	—	II, III	5B1	wsnp_Ex_c2582_4804223	Tdurum_contig10268_1000	153	3.5364	0.3448	12.2996	152.5-153.5	—
MMLPT	*AQ.MMLPT.ndsu.5B*	—	I, VII	5B1	RAC875_c33933_350	JD_c9261_426	49	3.7684	−0.2447	7.2642	48.5-63.5	—
BMT	*AQ.BMT.ndsu.5D*	MMLPT	IV, V, X	5D1	BS00110953_51	Excalibur_c16573_197	18	4.5987	−0.0698	3.4365	9.5-19.5	—
MMLPT	*AQ.MMLPT.ndsu.5D*	BMT	IV, VI, VIII, VIIII, X	5D1	BS00110953_51	Excalibur_c16573_197	19	7.4008	−0.1963	15.3925	12.5-19.5	—
BLV	*AQ.BLV.ndsu.6B*	CTCL	II, III	6B1	BobWhite_c10140_297	BobWhite_c8571_699	52	6.1493	5.56	15.4305	51.5-52.5	—
CBCL	*AQ.CBCL.ndsu.6B*	—	II, X	6B1	CAP8_c1678_709	Kukri_c23433_416	46	4.4927	0.0378	3.1303	44.5-46.5	Groos *et al.* (2007)
CTCL	*AQ.CTCL.ndsu.6B.1*	BLV	III	6B1	BobWhite_c10140_297	BobWhite_c8571_699	52	5.5319	0.2905	16.3676	51.5-52.5	—
FE	*AQ.FE.ndsu.6B*	—	II, IV, X	6B1	BobWhite_c30500_527	Excalibur_c31379_71	95	5.4465	−0.3753	8.367	94.5-95.5	—
BA	*AQ.BA.ndsu.6D*	—	II, VIII	6D1	wsnp_Ex_c23383_32628864	BobWhite_c13435_700	43	4.6326	−1.3204	3.7619	41.5-44.5	—
BLV	*AQ.BLV.ndsu.7A.1*	—	IV, X	7A1	Excalibur_rep_c109881_701	Tdurum_contig16202_319	59	4.5713	1.439	8.3454	58.5-59.5	—
BLV	*AQ.BLV.ndsu.7A.2*	—	IV, X	7A1	RAC875_c9012_276	BobWhite_c15497_199	118	6.5815	1.7646	12.6133	116.5-118.5	—
BMT	*AQ.BMT.ndsu.7A*	—	IV, X	7A1	Excalibur_c44794_122	RAC875_c55351_223	5	5.5287	0.0764	4.1206	1.5-6.5	—
CTCL	*AQ.CTCL.ndsu.7A*	MMLPV	III, X	7A1	Excalibur_c33589_373	RAC875_rep_c111778_387	86	5.6857	0.016	15.7116	85.5-86.5	—
GPC	*AQ.GPC.ndsu.7A.1*	MMLPT	II	7A1	BobWhite_c23261_226	BS00022970_51	24	4.2443	0.1848	6.5514	22.5-24.5	—
MMLPT	*AQ.MMLPT.ndsu.7A.1*	GPC	VIII	7A1	BobWhite_c23261_226	BS00022970_51	24	3.6069	−0.2228	5.9423	23.5-24.5	—
MMLPV	*AQ.MMLPV.ndsu.7A.1*	CTCL	IV	7A1	Excalibur_c33589_373	RAC875_rep_c111778_387	86	4.1338	1.8898	11.2755	84.5-86.5	—
GPC	*AQ.GPC.ndsu.7A*	—	IV, VII, VIII, X	7A2	BobWhite_c55693_396	BS00023003_51	16	4.6188	0.1507	7.1353	15.5-17	Christov *et al.* 2007
BLV	*AQ.BLV.ndsu.7B*	—	V, X	7B1	BobWhite_c41356_62	wsnp_CAP7_c44_26549	33	3.7635	3.4251	10.7091	31.5-39.5	—
MMLPT	*AQ.MMLPT.ndsu.7B*	—	I, III	7B1	BobWhite_c44404_312	CAP12_c1816_325	42	4.3413	−0.3644	3.6894	41.5-50.5	—
BMT	*AQ.BMT.ndsu.7D*	—	I,V	7D1	Kukri_c23468_590	Kukri_c16416_647	12	3.4443	0.1253	4.8285	7.5-13.5	—
FE	*AQ.FE.ndsu.7D*	—	IV, VI	7D2	RAC875_c39217_314	Excalibur_c16580_388	1	3.518	0.7611	11.1963	0-3.5	—
DO	*AQ.DO.ndsu.7D*	—	VI, X	7D3	wsnp_BE490643D_Ta_2_1	BobWhite_rep_c65034_450	71	4.1343	−0.0572	13.6687	70.5-72.5	—
MMLPT	*AQ.MMLPT.ndsu.7D*	—	I, III	7D3	IAAV6265	BobWhite_c7263_337	27	3.544	0.315	3.0233	25.5-32.5	—

a GPC: grain protein content, BMT: bake mixing time, BA: baking absorption, BLV: bread loaf volume, CBCL: crumb color, CTCL: crust color, FE: flour extraction rate, MIXOPA: the general mixograph pattern, MELS: mixograph envelope left slope, MERS: mixograph envelope right slope, MMLPT: mixograph MID line peak time, MMLPV: mixograph MID line peak value, MMLTV: mixograph MID line time * value, MMLPW: mixograph MID line peak width, MMLPI: mixograph MID line peak integral, DO: dough character.

b I: Prosper 2012, II: Carrington 2012, III: Casselton 20012, IV: Prosper 2013, V: Carrington 2013, VI: Minot 2013, VII: Prosper 2014, VIII: Carrington 2014, VIII: Minot 2014, X: BLUP values across all locations.

c centimorgan.

d Log of the Odds.

e Phenotypic variation.

**Table 6:** Digenic epistatic QTL (DE-QTL) detected for end-use quality traits in a bread wheat (*Triticum aestivum* L.) RIL population derived from a cross between Glenn (PI-639273) and Traverse (PI-642780)

Trait*^a^*	DE-QTL Name	Env.*^b^*	Other associated traits	Chrom.1 name	Position1*^c^*	Left Marker1	Right Marker1	Chrom.2 name	Position2*^c^*	Left Marker2	Right Marker2	Associated A-QTL	LOD*^d^*	PV(%)*^e^*	Additive by Additive Effects
BA	*DEQ.BA.ndsu.1A1/1A1*	II, VI, X	—	1A1	5	Kukri_c13513_759	RAC875_c50463_808	1A1	30	RFL_Contig1703_695	Excalibur_rep_c92985_618	—	3.86	6.94	1.28
BMT	*DEQ.BMT.ndsu.1A1/1A1*	VI, X	—	1A1	0	Kukri_c13513_759	RAC875_c50463_808	1A1	120	BobWhite_c27541_67	IAAV2729	—	3.64	2.10	0.06
BMT	*DEQ.BMT.ndsu.1A1/4D1*	V, X	—	1A1	120	BobWhite_c27541_67	IAAV2729	4D1	0	wsnp_JD_rep_c51623_35119179	Ra_c350_837	AQ.BA.ndsu.4D.1	3.58	1.90	−0.12
MMLPT	*DEQ.MMLPT.ndsu.1A1/4D1*	I, VIIII, X	—	1A1	5	Kukri_c13513_759	RAC875_c50463_808	4D1	0	wsnp_JD_rep_c51623_35119179	Ra_c350_837	AQ.BA.ndsu.4D.1	4.54	2.32	−0.22
MMLPW	*DEQ.MMLPW.ndsu.1A1/5A1*	II, X	—	1A1	35	RFL_Contig1703_695	Excalibur_rep_c92985_618	5A1	60	IAAV3916	RAC875_c54693_298	—	5.08	2.56	−1.20
MIXOPA	*DEQ.MIXOPA.ndsu.1A1/7A1*	VIII, X	MMLTV	1A1	125	BobWhite_c27541_67	IAAV2729	7A1	170	wsnp_Ex_c6354_11053460	BS00053365_51	—	4.87	1.27	0.15
MMLTV	*DEQ.MMLTV.ndsu.1A1/7A1*	VIII, VIIII	MIXOPA	1A1	130	BobWhite_c27541_67	IAAV2729	7A1	180	Excalibur_c48973_1688	IACX6080	—	3.60	2.23	2.13
MMLPW	*DEQ.MMLPW.ndsu.1A1/7B1*	I, X	—	1A1	0	Kukri_c13513_759	RAC875_c50463_808	7B1	0	Tdurum_contig57324_104	Excalibur_c21252_227	—	3.51	1.35	0.81
GPC	*DEQ.GPC.ndsu.1A1/7D3*	II, V	MERS	1A1	15	Excalibur_c5139_198	wsnp_Ex_c1358_2601510	7D3	20	Kukri_c37793_135	Kukri_c9804_462	—	4.73	1.30	−0.30
GPC	*DEQ.GPC.ndsu.1A1/7D3*	I, X	—	1A1	30	RFL_Contig1703_695	Excalibur_rep_c92985_618	7D3	25	IAAV6265	BobWhite_c7263_337	—	3.51	1.90	−0.13
MERS	*DEQ.MERS.ndsu.1A1/7D3*	V, X	GPC	1A1	15	Excalibur_c5139_198	wsnp_Ex_c1358_2601510	7D3	20	Kukri_c37793_135	Kukri_c9804_462	—	5.74	3.16	1.30
MMLPV	*DEQ.MMLPV.ndsu.1B1/7B1*	VII, VIII	MMLTV	1B1	0	RAC875_c4385_1628	wsnp_Ra_c23758_33291657	7B1	50	CAP12_c1816_325	BobWhite_c14812_828	—	3.88	8.15	2.56
MMLTV	*DEQ.MMLTV.ndsu.1B1/7B1*	VII, VIII	MMLPV	1B1	5	RAC875_c4385_1628	wsnp_Ra_c23758_33291657	7B1	45	CAP12_c1816_325	BobWhite_c14812_828	—	4.80	3.46	3.20
MMLPT	*DEQ.MMLPT.ndsu.1D1/5D1*	V, X	—	1D1	20	RAC875_c16352_594	CAP8_c2401_433	5D1	0	wsnp_Ku_c44483_51751682	wsnp_JD_c825_1223454	—	3.96	1.90	0.33
MMLPI	*DEQ.MMLPI.ndsu.2A1/2B1*	IV, VIIII	—	2A1	5	Excalibur_c51876_189	wsnp_Ku_c10302_17079851	2B1	30	TA002233-0872	Ku_c36209_204	—	4.06	0.92	7.74
MMLPT	*DEQ.MMLPT.ndsu.2A1/2B2*	I, II, X	—	2A1	10	wsnp_JD_rep_c48914_33168544	wsnp_Ex_rep_c102538_87682273	2B2	20	GENE-0592_352	BS00064658_51	—	5.59	1.87	−0.61
FE	*DEQ.FE.ndsu.2A1/3A2*	II, X	—	2A1	105	BobWhite_rep_c50285_616	Tdurum_contig67827_98	3A2	0	Ex_c35861_1382	Tdurum_contig42150_3190	—	3.35	1.72	−0.27
MIXOPA	*DEQ.MIXOPA.ndsu.2A1/3A2*	VIIII, X	—	2A1	45	Excalibur_c65910_246	RAC875_c81899_216	3A2	45	BobWhite_c38444_238	RAC875_c15109_106	AQ.BMT.ndsu.3A	3.75	1.20	−0.41
MIXOPA	*DEQ.MIXOPA.ndsu.2A1/5B*	VIII, X	—	2A1	115	IAAV880	CAP12_c575_105	5B	225	GENE-2471_259	Kukri_c9285_762	—	4.20	2.59	−0.31
MMLPT	*DEQ.MMLPT.ndsu.2A1/6D1*	I, X	—	2A1	0	wsnp_Ex_c5412_9565527	Ra_c10616_265	6D1	35	wsnp_Ex_c23383_32628864	BobWhite_c13435_700	AQ.BA.ndsu.6D	4.13	1.91	−0.63
MERS	*DEQ.MERS.ndsu.2A1/7A1*	IV, X	—	2A1	125	CAP8_c3129_381	Tdurum_contig92425_3144	7A1	185	Excalibur_c1142_724	Tdurum_contig54832_139	—	4.04	2.71	0.42
MMLPT	*DEQ.MMLPT.ndsu.2A2/4B1*	II, III, VII, VIIII	—	2A2	0	Excalibur_c29231_932	RAC875_c8069_1709	4B1	55	wsnp_Ex_c26285_35532440	RAC875_rep_c119568_203	—	5.00	2.19	−0.21
MELS	*DEQ.MELS.ndsu.2B1/2B2*	I, II	—	2B1	10	BobWhite_c19554_544	Kukri_c9785_1557	2B2	95	BobWhite_c23046_293	wsnp_Ex_c3695_6740339	—	5.49	1.87	−6.74
BMT	*DEQ.BMT.ndsu.2B2/1D1*	VI, VIII	—	2B2	15	BobWhite_rep_c64429_660	Kukri_c53810_315	1D1	60	CAP8_c1305_148	BS00022168_51	—	3.37	0.89	−0.13
MMLPT	*DEQ.MMLPT.ndsu.2B2/1D1*	II, VI	—	2B2	100	BobWhite_c23046_293	wsnp_Ex_c3695_6740339	1D1	45	CAP8_c1305_148	BS00022168_51	—	4.37	1.99	−0.74
FE	*DEQ.FE.ndsu.2B2/2D2*	I, X	—	2B2	170	Excalibur_c15671_87	Excalibur_c29221_311	2D2	5	Kukri_c9478_2764	Kukri_c65380_490	—	3.11	1.75	0.27
BMT	*DEQ.BMT.ndsu.2B2/5B*	IV, VI	—	2B2	100	BobWhite_c23046_293	wsnp_Ex_c3695_6740339	5B	30	BS00064297_51	wsnp_BE499835B_Ta_2_5	AQ.GPC.ndsu.5B	8.45	2.50	−0.20
GPC	*DEQ.GPC.ndsu.2B2/5B*	II, X	—	2B2	0	BS00070900_51	GENE-1343_315	5B	125	Kukri_c34173_169	wsnp_Ku_c3201_5970486	—	5.09	1.51	−0.28
BMT	*DEQ.BMT.ndsu.2B2/6B1*	V, X	—	2B2	25	GENE-0592_352	BS00064658_51	6B1	135	wsnp_Ex_c9038_15058444	Tdurum_contig43335_1397	—	4.27	3.25	−0.16
FE	*DEQ.FE.ndsu.2B2/7D1*	II, X	—	2B2	65	Excalibur_c45094_602	BS00040959_51	7D1	15	wsnp_Ex_c17914_26681837	RAC875_c11933_885	—	4.13	2.45	−0.31
MMLPT	*DEQ.MMLPT.ndsu.2B2/7D3*	V, X	—	2B2	50	RFL_Contig996_818	Tdurum_contig30989_79	7D3	15	Kukri_c37793_135	Kukri_c9804_462	—	3.44	1.82	0.17
MMLPT	*DEQ.MMLPT.ndsu.3A1/2D1*	IV, VIIII, X	—	3A1	0	Tdurum_contig74920_757	CAP8_rep_c3652_80	2D1	10	RAC875_c110838_423	Kukri_c12032_508	—	4.35	1.69	−0.17
MMLPT	*DEQ.MMLPT.ndsu.3A1/6A1*	I, II	—	3A1	65	BS00077819_51	Kukri_c51666_401	6A1	55	BobWhite_c1131_328	Excalibur_c29639_65	—	3.52	2.33	0.33
MMLPT	*DEQ.MMLPT.ndsu.3A1/7A1*	I, IV	—	3A1	50	TA002540-0938	RAC875_c52195_324	7A1	45	BS00065020_51	tplb0024a09_2106	—	4.03	1.31	0.51
GPC	*DEQ.GPC.ndsu.3B1/2D2*	VII, X	—	3B1	45	wsnp_Ex_c26128_35374652	Excalibur_c45968_83	2D2	10	Excalibur_rep_c104620_183	wsnp_BE426620D_Ta_2_2	—	5.42	2.23	0.15
BMT	*DEQ.BMT.ndsu.3B2/4B1*	V, X	—	3B2	30	CAP12_c1468_114	JD_c37202_67	4B1	45	wsnp_CAP12_c1101_569783	BS00042105_51	—	5.54	2.13	0.07
FE	*DEQ.FE.ndsu.3B3/4B1*	II, X	—	3B3	5	BS00087695_51	BS00003884_51	4B1	100	wsnp_Ra_c10988_17932922	RAC875_rep_c82932_428	—	3.41	1.92	0.29
BMT	*DEQ.BMT.ndsu.3B4/5B*	II, VIII	—	3B4	5	BS00022154_51	wsnp_Ex_rep_c66766_65123941	5B	180	Excalibur_c12395_467	wsnp_Ex_c32488_41134388	—	3.25	1.44	0.15
MIXOPA	*DEQ.MIXOPA.ndsu.4A1/1B1*	I, X	—	4A1	10	BS00035307_51	RAC875_c16277_737	1B1	60	RAC875_c61512_173	wsnp_Ex_c9091_15135511	—	3.56	1.21	−0.15
MERS	*DEQ.MERS.ndsu.4A1/1D1*	IV, VI, X	—	4A1	95	wsnp_Ku_c4924_8816643	Tdurum_contig42526_994	1D1	10	Excalibur_c35316_137	RAC875_c16352_594	—	5.03	5.59	1.69
MMLPI	*DEQ.MMLPI.ndsu.4A1/2D2*	IV, VI	—	4A1	55	RFL_Contig5998_745	RAC875_c65221_438	2D2	5	Kukri_c9478_2764	Kukri_c65380_490	—	4.78	1.44	11.39
MMLPT	*DEQ.MMLPT.ndsu.4A1/5A1*	I, III, IV, V	—	4A1	90	Tdurum_contig47148_651	RAC875_c25124_182	5A1	30	Kukri_c28555_114	wsnp_Ku_c18023_27232712	AQ.BLV.ndsu.5A	4.19	1.66	0.55
GPC	*DEQ.GPC.ndsu.4A1/6D2*	III,VIIII	—	4A1	85	Ex_c66324_1151	wsnp_Ex_c5470_9657856	6D2	0	BS00022523_51	Kukri_rep_c105352_281	—	3.29	1.04	−0.19
BMT	*DEQ.BMT.ndsu.4A1/7B1*	I, VI	—	4A1	35	wsnp_Ex_c22913_32130617	CAP12_c2677_138	7B1	40	BobWhite_c41356_62	wsnp_CAP7_c44_26549	—	4.63	1.03	−0.20
GPC	*DEQ.GPC.ndsu.4A1/7B1*	VII, VIIII	—	4A1	5	BS00035307_51	RAC875_c16277_737	7B1	80	BobWhite_c6580_361	wsnp_Ex_c10550_17231294	—	3.60	3.49	0.30
MMLPW	*DEQ.MMLPW.ndsu.4A1/7B1*	VIIII, X	—	4A1	80	Kukri_c27874_515	Ex_c66324_1151	7B1	5	Excalibur_c21252_227	Excalibur_c8486_471	—	3.97	1.63	0.30
MMLPT	*DEQ.MMLPT.ndsu.4B1/2D1*	IV, VII	—	4B1	70	Excalibur_c39876_403	Kukri_c19909_733	2D1	10	RAC875_c110838_423	Kukri_c12032_508	—	4.03	1.00	0.18
BMT	*DEQ.BMT.ndsu.4B1/5B*	V, VII, X	—	4B1	90	wsnp_Ex_c15490_23776560	IAAV8499	5B	0	BS00032003_51	BS00064297_51	AQ.GPC.ndsu.5B	5.65	2.58	0.20
MMLTV	*DEQ.MMLTV.ndsu.4B1/5D1*	VII, X	—	4B1	60	RAC875_rep_c119568_203	Tdurum_contig59914_323	5D1	20	wsnp_Ex_c5185_9189184	D_GDS7LZN02F4FP5_176	—	3.70	1.96	2.38
FE	*DEQ.FE.ndsu.5A1/1D1*	II, IV, VI, VII	—	5A1	35	Kukri_c28555_114	wsnp_Ku_c18023_27232712	1D1	25	RAC875_c16352_594	CAP8_c2401_433	AQ.BLV.ndsu.5A	4.65	3.84	1.07
MMLPI	*DEQ.MMLPI.ndsu.5A1/5A2*	IV, VI	—	5A1	35	Kukri_c28555_114	wsnp_Ku_c18023_27232712	5A2	10	BS00022683_51	BobWhite_c17440_130	AQ.BLV.ndsu.5A	4.61	1.85	−13.09
MMLPI	*DEQ.MMLPI.ndsu.5A1/7B1*	IV, VI, X	—	5A1	20	wsnp_Ex_c31672_40435001	Kukri_c28555_114	7B1	65	Kukri_c18749_968	Tdurum_contig12064_92	—	3.58	1.42	11.23
MMLPI	*DEQ.MMLPI.ndsu.5A1/7D3*	IV, VIIII	MMLPT, MMLTV	5A1	75	BS00020605_51	BobWhite_c11539_336	7D3	50	Tdurum_contig46368_632	RAC875_c68368_99	—	4.72	1.52	−9.66
MMLPT	*DEQ.MMLPT.ndsu.5A1/7D3*	I, IV	MMLTV, MMLPI	5A1	70	BS00020605_51	BobWhite_c11539_336	7D3	45	Tdurum_contig46368_632	RAC875_c68368_99	—	4.69	1.63	−0.23
MMLTV	*DEQ.MMLTV.ndsu.5A1/7D3*	IV, X	MMLPT, MMLPI	5A1	70	BS00020605_51	BobWhite_c11539_336	7D3	55	Tdurum_contig46368_632	RAC875_c68368_99	—	3.17	2.98	−0.64
MMLPI	*DEQ.MMLPI.ndsu.5A2/7A1*	VI, X	—	5A2	25	Kukri_c41797_393	Ex_c19057_965	7A1	80	wsnp_Ex_c5939_10417052	wsnp_Ex_c39221_46569987	—	3.88	4.13	−4.30
GPC	*DEQ.GPC.ndsu.5A3/2B2*	I, X	—	5A3	5	BS00099534_51	Excalibur_c6714_246	2B2	5	IAAV5802	GENE-1676_1048	—	3.91	1.89	−0.16
MMLPT	*DEQ.MMLPT.ndsu.5A3/3B4*	III, VII,X	—	5A3	5	BS00099534_51	Excalibur_c6714_246	3B4	5	BS00022154_51	wsnp_Ex_rep_c66766_65123941	—	3.62	1.64	−0.15
BMT	*DEQ.BMT.ndsu.5B/2D1*	V, VII, X	—	5B	105	CAP12_c1419_574	RAC875_c14780_54	2D1	0	RAC875_c110838_423	Kukri_c12032_508	—	3.79	2.90	−0.07
GPC	*DEQ.GPC.ndsu.5B/6D1*	VI, VIII	—	5B	30	BS00064297_51	wsnp_BE499835B_Ta_2_5	6D1	45	wsnp_Ex_c23383_32628864	BobWhite_c13435_700	AQ.GPC.ndsu.5B x AQ.BA.ndsu.6D	5.73	0.79	0.98
MELS	*DEQ.MELS.ndsu.5B1/6B1*	I, X	—	5B1	170	BobWhite_rep_c50349_139	Kukri_c10508_755	6B1	100	BS00037933_51	BS00063217_51	—	3.86	1.51	−0.74
BMT	*DEQ.BMT.ndsu.5D1/6D1*	IV, X	—	5D1	15	BS00110953_51	Excalibur_c16573_197	6D1	35	wsnp_Ex_c23383_32628864	BobWhite_c13435_700	AQ.BMT.ndsu.5D x AQ.BA.ndsu.6D	3.98	1.64	0.17
MMLPT	*DEQ.MMLPT.ndsu.6A1/4B1*	IV, VI	—	6A1	5	RAC875_c32053_291	BobWhite_c44549_83	4B1	110	wsnp_Ku_c7838_13435765	Excalibur_c26571_370	—	4.43	0.77	0.40
MMLPT	*DEQ.MMLPT.ndsu.6A2/5B*	I, X	—	6A2	10	BS00110512_51	BS00065028_51	5B	40	BS00064297_51	wsnp_BE499835B_Ta_2_5	AQ.GPC.ndsu.5B	4.88	2.05	−0.59
GPC	*DEQ.GPC.ndsu.6B1/2D2*	II, VIIII	—	6B1	100	BS00037933_51	BS00063217_51	2D2	0	wsnp_RFL_Contig2659_2346243	RAC875_c78404_242	—	4.89	2.22	−0.18
BLV	*DEQ.BLV.ndsu.6D1/7D3*	II, X	—	6D1	5	BobWhite_c14066_403	Ra_c32572_334	7D3	20	Kukri_c37793_135	Kukri_c9804_462	—	4.09	3.37	1.43
MIXOPA	*DEQ.MIXOPA.ndsu.7A1/7B1*	VIIII, X	—	7A1	50	tplb0024a09_2106	Tdurum_contig98029_517	7B1	5	Excalibur_c21252_227	Excalibur_c8486_471	—	3.84	1.44	0.41
MMLPT	*DEQ.MMLPT.ndsu.7A1/7D1*	I, VII	—	7A1	65	wsnp_Ex_c13337_21022241	RAC875_c28842_99	7D1	20	BS00066128_51	BS00083421_51	—	4.04	2.20	−0.32
BMT	*DEQ.BMT.ndsu.7A1/7D3*	V, X	—	7A1	25	BS00106739_51	Excalibur_rep_c68458_1536	7D3	70	wsnp_BE490643D_Ta_2_1	BobWhite_rep_c65034_450	—	5.08	2.28	0.07
MMLPT	*DEQ.MMLPT.ndsu.7A1/7D3*	I, X	—	7A1	55	BS00011330_51	Tdurum_contig67992_238	7D3	75	BobWhite_rep_c65034_450	wsnp_CAP8_rep_c9647_4198594	—	4.32	1.81	−0.17
MIXOPA	*DEQ.MIXOPA.ndsu.7A2/7B1*	VIIII, X	—	7A2	10	Kukri_c40353_179	Excalibur_c59653_238	7B1	5	Excalibur_c21252_227	Excalibur_c8486_471	—	6.97	1.22	0.17
BMT	*DEQ.BMT.ndsu.7B1/7D2*	IV, VII	—	7B1	110	wsnp_Ra_c39394_47110214	BobWhite_c26534_532	7D2	5	Excalibur_c16580_388	Kukri_c19321_416	—	3.62	1.65	0.14
MMLPI	*DEQ.MMLPI.ndsu.7D1/7D3*	IV, VIIII	—	7D1	0	BS00051338_51	IAAV5917	7D3	40	BobWhite_c7263_337	Tdurum_contig46368_632	—	4.74	1.96	−13.80

a GPC: grain protein content, BMT: bake mixing time, BA: baking absorption, BLV: bread loaf volume, CBCL: crumb color, CTCL: crust color, FE: flour extraction rate, MIXOPA: the general mixograph pattern, MELS: mixograph envelope left slope, MERS: mixograph envelope right slope, MMLPT: mixograph MID line peak time, MMLPV: mixograph MID line peak value, MMLTV: mixograph MID line time * value, MMLPW: mixograph MID line peak width, MMLPI: mixograph MID line peak integral, DO: dough character.

b I: Prosper 2012, II: Carrington 2012, III: Casselton 20012, IV: Prosper 2013, V: Carrington 2013, VI: Minot 2013, VII: Prosper 2014, VIII: Carrington 2014, VIII: Minot 2014, X: BLUP values across all locations.

c centimorgan.

d Log of the Odds.

e Phenotypic variation.

### Quantitative Trait Loci for Grain Protein Content

**Figure 2:**
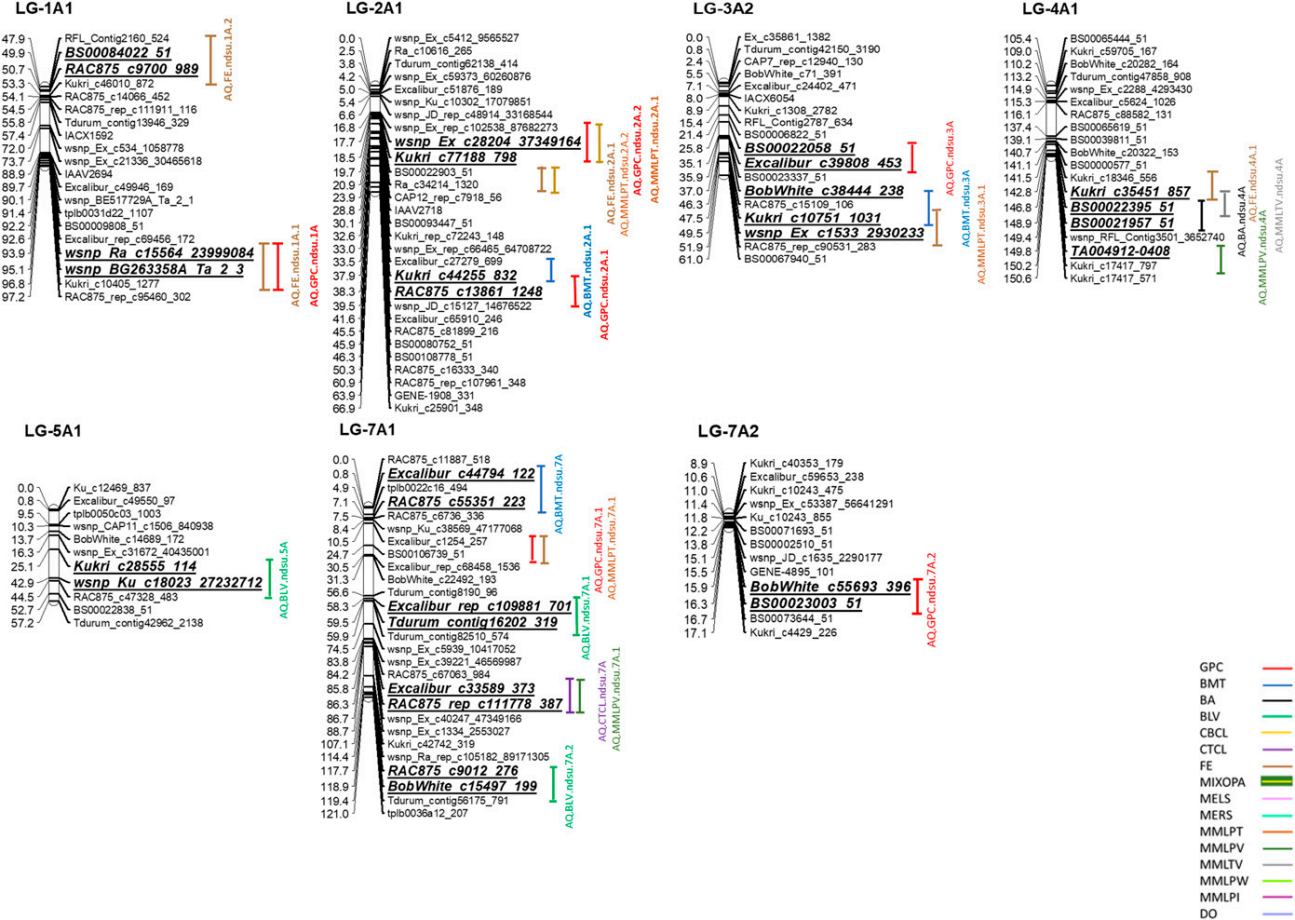

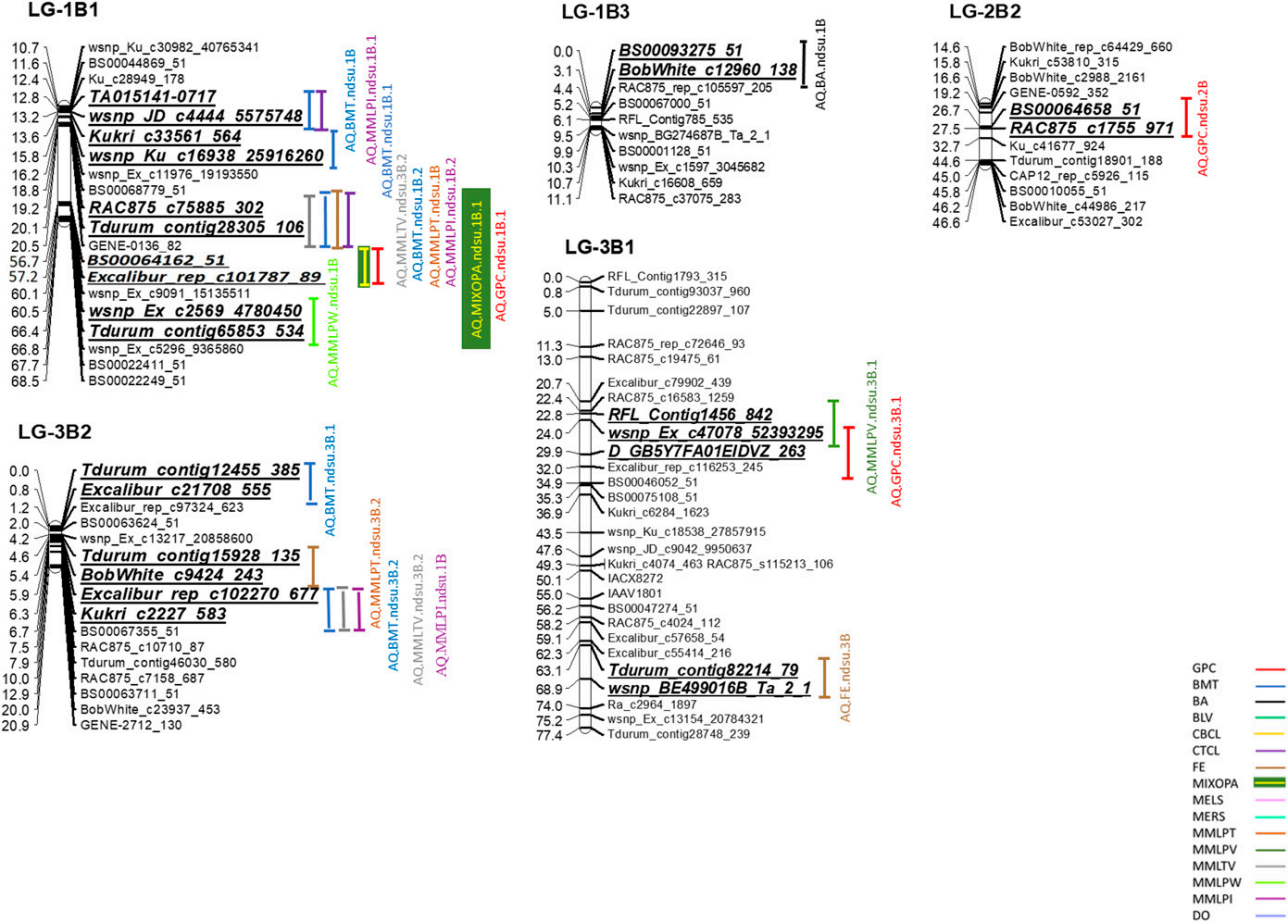

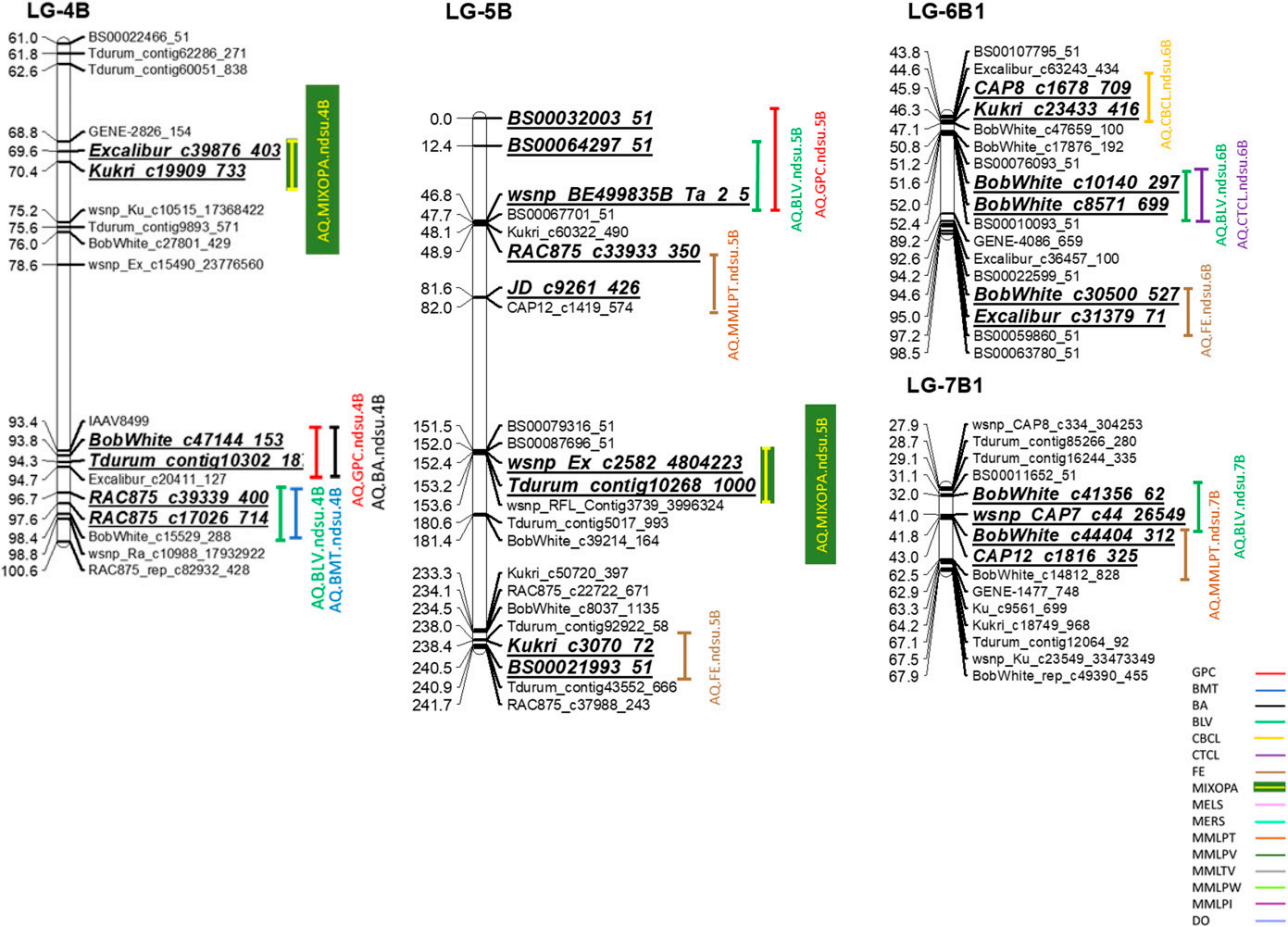

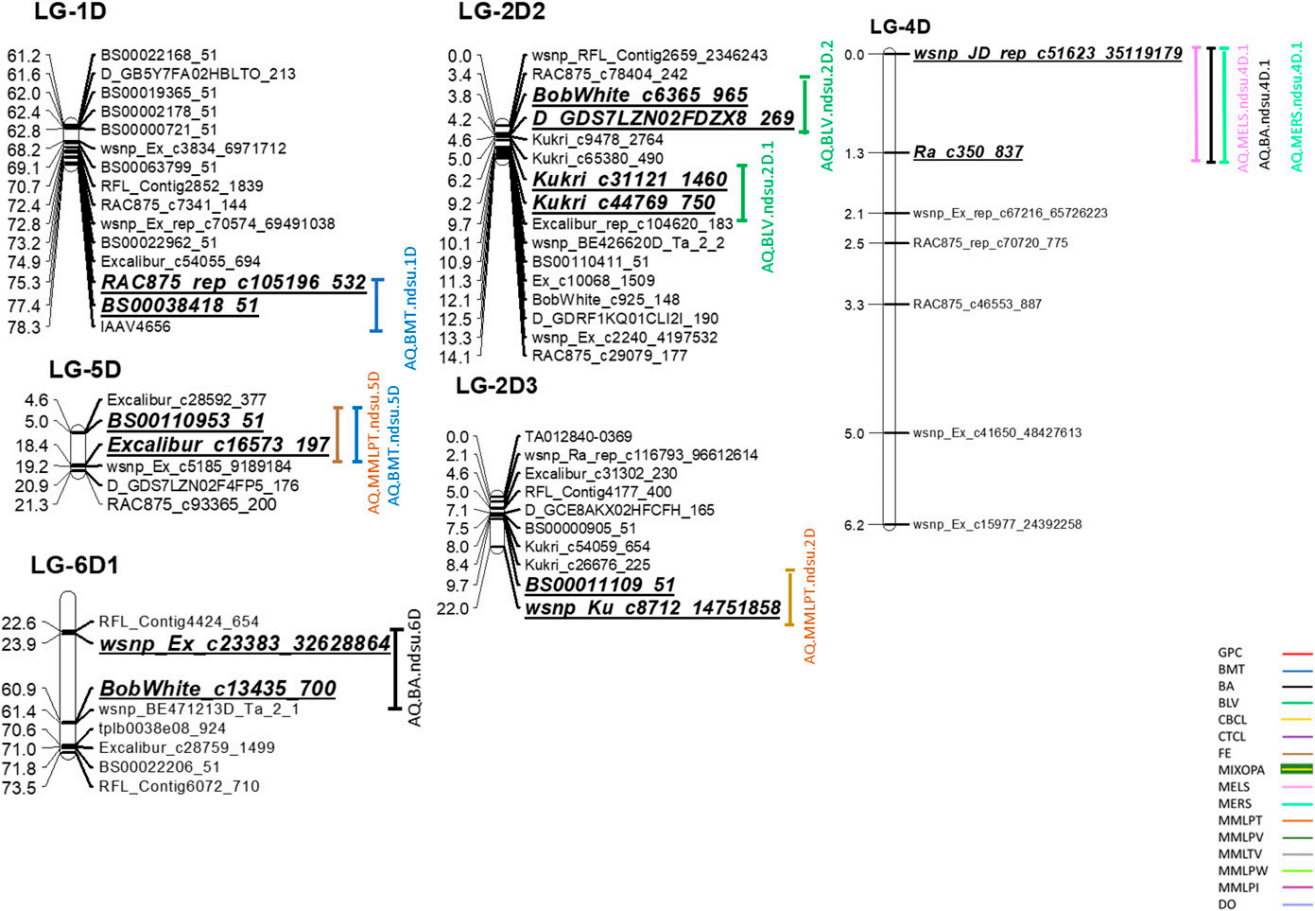

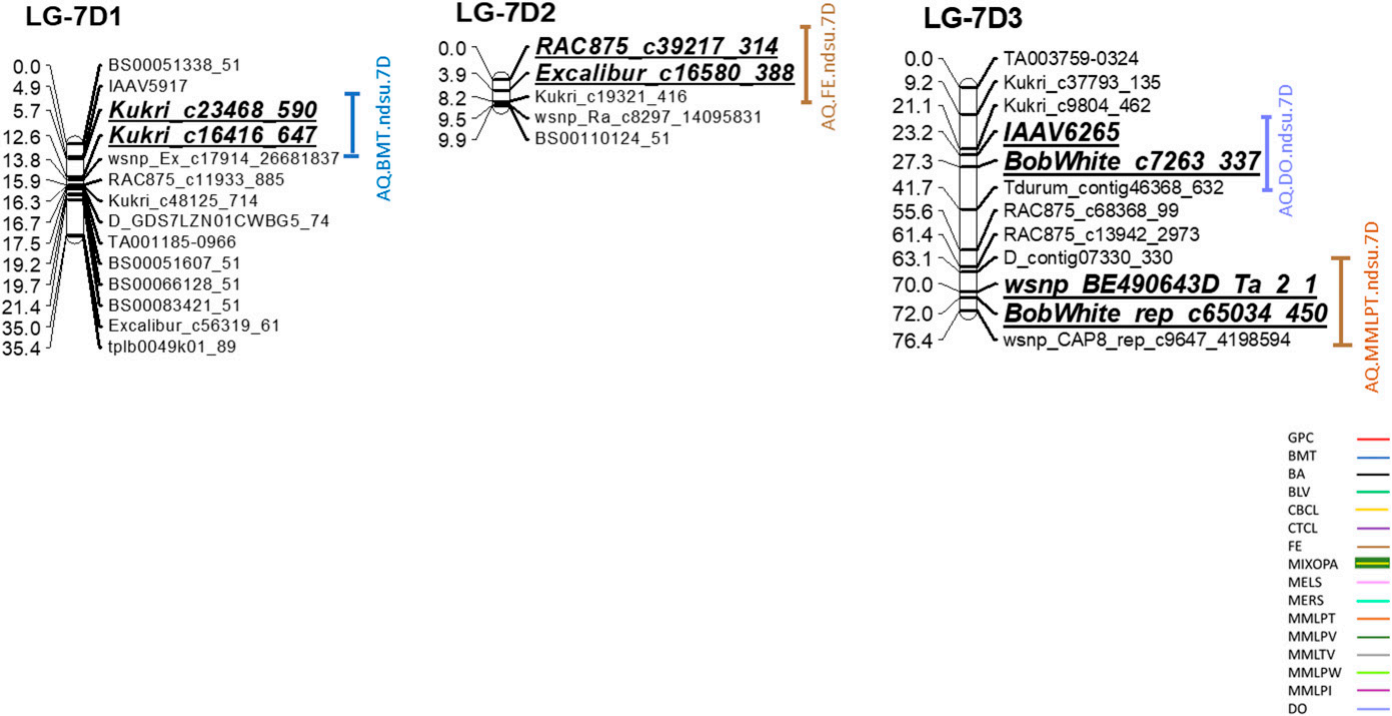
Additive and additive co-localized QTL for end-use quality traits in the Glenn × Traverse RIL population. QTL confidence intervals are indicated by vertical bars and bold and italic scripts.

A total of 11 A-QTL and 18 DE-QTL were detected for grain protein content (Table 5; Table 6; Figure 2). The 11 A-QTL were located on chromosomes /linkage groups 1A1, 1B1, 2A1, 2B2, 3A2, 3B1, 4B, 5B, and 7A2. No A-QTL was found on the D-genome for grain protein content in this study. Five A-QTL individually explained over 10% of PV and were considered major A-QTL. The major A-QTL were located on chromosomes/linkage groups 1A1, 2A1, 3B1, 4B, and 5B (Table 5; Figure 2). Three A-QTL were detected in more than three environments and were considered stable A-QTL. Two of these stable A-QTL, *AQ.GPC.ndsu.1A* and *AQ.GPC.ndsu.5B*, explained up to13.69% and 20.18% of PV for grain protein content, respectively, and were also considered major QTL. For this trait, both parental genotypes contributed positive alleles, although the majority of the alleles (including the three stable A-QTL) were contributed by the cultivar Glenn (Table 5; Figure 2). The QTL *AQ.GPC.ndsu.7A* showed sequence similarity with wheat HMGB1 mRNA for high mobility globular protein. Christov *et al.* (2007) suggested the wheat HMGB1 protein may have a specific function as a general regulator of gene expression during cold acclimation in wheat.

The results of digenic epistatic effects for grain protein content are shown in Table 6. The accumulated contribution of these nine epistatic interactions for grain protein content was ∼16.38%. These DE-QTL were located on pairs of linkage groups 1A1/7D3, 1A1/7D3, 2B2/5B1, 3B1/2D2, 4A1/7B1, 4A1/6D2, 5A3/2B2, 5B/6D1, and 6B1/2D2. Unlike A-QTL, DE-QTL for grain protein content were identified on the D-genome. The majority of these DE-QTL showed negative values for digenic epistatic effects indicating the positive effects of recombinant genotypic combinations on grain protein content. The *AQ.GPC.ndsu.5B* had the most important main effect on grain protein content, and the *AQ.BA.ndsu.6D* had a significant main effect on BA; the epistatic interaction between these A-QTL had a positive effect on grain protein content. The parental genotypic combinations increased grain protein content through this interaction (Table 6).

### Quantitative Trait Loci for Flour Extraction and Mixograph-related Parameters

A total of 32 A-QTL and 51 DE-QTL were identified for flour extraction and mixograph-related parameters (Table 5; Table 6; Figure 2). These 32 A-QTL were located across all 21 wheat chromosomes except chromosomes 1D, 2B, 3D, 5A, 6A, and 6D. A total of 19 A-QTL individually explained more than 10% of PV and were considered major A-QTL. Out of these A-QTL, five stable A-QTL were found for these traits, one stable A-QTL for flour extraction (*AQ.FE.ndsu.3B*) and four stable A-QTL for mixograph MID line peak time (*AQ.MMLPT.ndsu.1B*, *AQ.MMLPT.ndsu.5D*, *AQ.MMLPT.ndsu.3B.2*, and *AQ.MMLPT.ndsu.2D*). For all of these stable A-QTL, except the *AQ.MMLPT.ndsu.1B*, the alleles were contributed through the Traverse cultivar. The *AQ.MMLPT.ndsu.1B* A-QTL was identified in six out of nine environments and explained up to 24.35% of PV for MMPLT. This A-QTL was considered the most stable A-QTL, which had the highest effect on MMLPT (Table 5).

The results of DE-QTL for flour extraction and mixograph-related parameters are shown in Table 6. A total of 49 DE-QTL were detected on all wheat chromosomes expect chromosome 3D. The individual epistatic interactions explained ∼0.77% to ∼8.15% of PV for flour extraction and mixograph parameters. Three stable digenic epistatic interactions were found for these traits: one DE-QTL (*DEQ.FE.ndsu.5A1/1D1*) for flour extraction and two DE-QTL (*DEQ.MMLPT.ndsu.2A2/4B1* and *DEQ.MMLPT.ndsu.4A1/5A1*) for mixograph MID line peak time. The *DEQ.FE.ndsu.5A1/1D1* DE-QTL explained only up to 3.84% of PV for flour extraction. The parental genotypic combinations of this DE-QTL had a positive effect on the increase of flour extraction. The *DEQ.MMLPT.ndsu.2A2/4B1* and *DEQ.MMLPT.ndsu.4A1/5A1* DE-QTL explained only up to 2.19% and 1.66% of PV for mixograph MID line peak time, respectively. The parental genotypic combinations increased MMPLT through the *DEQ.MMLPT.ndsu.4A1/5A1* stable DE-QTL, whereas recombinant genotypic combinations increased MMPLT through the *DEQ.MMLPT.ndsu.2A2/4B1* stable DE-QTL. Overall, both parental and recombinant genotypic combinations almost equally contributed to the increase of flour extraction and improvement of the mixograph-related parameters (Table 6).

### Quantitative Trait Loci for Baking Properties

A total of 31 A-QTL and 15 DE-QTL were detected for baking-related properties in this study (Table 5; Table 6; Figure 2). These 31 A-QTL individually explained ∼2.14% to ∼28.06% of PV for the associated traits. These A-QTL were located on 17 wheat chromosomes excluding 1A, 2B, 3D, and 6A. A total of 19 major A-QTL with PV values over 10% were found for the baking-related properties. Three stable A-QTL were identified in this study: two A-QTL for baking absorption (*AQ.BA.ndsu.4D.1* and *AQ.BA.ndsu.1B*) and one A-QTL (*AQ.BMT.ndsu.5D*) for bake-mixing time. Although the Glenn cultivar contributed over 60% of the desirable alleles for the baking-related properties in this study, the cultivar Traverse contributed the desirable alleles for these three stable A-QTL. The *AQ.BA.ndsu.4D.1* stable A-QTL associated with baking absorption had the highest PV (∼28.06%) for end-use quality traits in this study (Table 5).

The results of digenic epistatic interactions for the baking-related properties are presented in Table 6. Out of the six baking-related properties evaluated in this study, digenic epistatic effects were only identified for baking absorption, bread loaf volume, and bake-mixing time traits with one, one, and 13 digenic epistatic interactions, respectively. The DE-QTL, *DEQ.BA.ndsu.1A1/1A1* and *DEQ.BLV.ndsu.6D1/7D3*, explained ∼6.94% and ∼3.37% of PV for baking absorption and bread loaf volume, respectively. The accumulated contribution of the 13 DE-QTL for bake-mixing time was ∼26.29%. Both parental and recombinant genotypic combinations contributed to the increase of bake-mixing time, whereas only the parental genotypic combinations had positive effects on baking absorption and BLV (Table 6).

### Co-Localized Quantitative Trait Loci

A total of 19 additive co-localized (closely linked or pleiotropic) QTL, and four epistatic co-localized QTL were found in this study (Table 5; Table 6; Figure 2). These 19 additive co-localized QTL were mainly located on the A- and B-genomes (Table 5; Figure 2). Positive pleiotropy was shown in 14 out of 19 additive co-localized QTL, where the additive effects of a locus on multiple traits were of the same sign. In contrast, negative pleiotropic effects were observed for five co-localized QTL on chromosomes/linkage groups 1A1, 2A1, 2A1, 4A, and 4D harboring major A-QTL, respectively, for grain protein content and flour extraction; grain protein content and bake-mixing time; grain protein content and mixograph MID line peak time; flour extraction, mixograph MID line time * value, and baking absorption; and mixograph envelope left slope, mixograph envelope right slope, and baking absorption. Overall, approximately 63% of A-QTL with close linkage or pleiotropic effects on the integrated set of traits (Table 5; Figure 2) were considered major A-QTL. Additive co-localized QTL for the end-use quality traits are shown in more detail in Table 5.

In addition to additive co-localized QTL, four epistatic co-localized QTL (“epistatic pleiotropy,” Wolf *et al.*, 2005) were identified in this study (Table 6). These epistatic co-localized QTL were located on pairs of linkage groups 1A1/7A1, 5A1/7D3, 1A1/7D3, and 1B1/7B1 associated with general mixograph pattern and mixograph MID line time * value; mixograph MID line peak time, mixograph MID line peak integral, and mixograph MID line time * value; grain protein content and mixograph envelope right slope; and mixograph MID line peak value and mixograph MID line time * value, respectively (Table 6). All epistatic co-localized QTL except one (1A1/7D3 for the integrated set of grain protein content and mixograph envelope right slope traits) showed positive pleiotropic effects (Table 6).

## Discussion

### Phenotypic Evaluation

It is well documented that end-use quality traits in wheat are complex and are influenced by a combination of environmental conditions and genetic factors (Rousset *et al.* 1992; Peterson *et al.* 1998; Tsilo *et al.* 2011; Simons *et al.* 2012). The power and accuracy of QTL detection are highly dependent on choosing the parental lines (Jansen *et al.* 2001). In other words, power of accuracy depend on allelic polymorphism and phenotypic variation between parental lines. In the current study, the RIL population was developed from a cross between Glenn (PI 639273) and Traverse (PI 642780). Glenn has excellent end-use quality characteristics. By comparison, Traverse has a high grain yield but poor end-use quality characteristics. As expected, our results showed significantly different values between the parental lines for most of the end-use quality traits. The RIL population showed continuous variation and transgressive segregation for all the end-use quality characteristics, suggesting the polygenetic inheritance and contribution, particularly of positive alleles for the end-use quality traits by both parental lines.

Our results showed a wide range of broad-sense heritability (0.23 – 0.77) for mixograph-related parameters, suggesting environmental effects had a wide range of influences on the phenotypic values of the mixograph-related parameters. These results were in agreement with those of Patil *et al.* (2009), who also reported a wide heritability range of 0.17 to 0.96 for mixograph-relative parameters. In contrast to our results, Tsilo *et al.* (2011) and Prashant *et al.* (2015) found high broad-sense heritability for most of the end-use quality traits in wheat. Similarly, the current study, Echeverry-Solarte *et al.* (2015) reported very high broad-sense heritability for flour extraction and MMLPT.

The genetic and Pearson correlation analyses revealed most of the end-use quality traits were associated with each other. Several previous studies have also reported similar results (Patil *et al.* 2009; Tsilo *et al.* 2011; Prashant *et al.* (2015); Echeverry-Solarte *et al.* 2015). Our results showed differences between genetic and phenotypic correlation coefficients for end-use quality traits. These differences could be due to low heritability values for these traits as was reported by Hill and Thompson (1978). Notably, although there were differences between the genetic and phenotypic correlation coefficients, the pattern and magnitude of these coefficients were similar. These similarities suggest the phenotypic correlation could be a fair estimate of the genetic correlation for end-use quality traits in wheat.

### High-Density Linkage Map

Genetic linkage maps have played important roles in detecting QTL, MAS, cloning genes, and genome structure analysis (Maccaferri *et al.* 2014; Jin *et al.* 2016). In the present study, the wheat Illumina 90K iSelect assay was used to genotype Glenn and Traverse and all 127 RILs derived from these two parents. Our study resulted in a much higher genome coverage and resolution compared to the most of the previous genetic linkage maps for the genetic dissection of end-use quality traits in wheat (Groos *et al.* 2003; Echeverry-Solarte *et al.* 2015; Boehm *et al.* 2017). Marker density of 0.33 cM between any two markers indicated a significant improvement over earlier genetic maps developed with either microsatellite markers (Tsilo *et al.* 2010; Simons *et al.* 2012), DArT markers (Echeverry-Solarte *et al.* 2015), or SNP makers (Boehm *et al.* 2017). The genetic map length of 2,644.82 cM improved significantly the genome coverage compared to the other developed map for the genetic analysis of end-use quality traits in wheat using the wheat Illumina 90K iSelect assay (Boehm *et al.* 2017), where the map size was 1813.4 cM.

Several large genetic gaps, in the framework of our linkage map, led us to split most of the chromosomes into more than one linkage group. These genetic gaps could be due to the following reasons: First, the genotyping for the RIL population was based solely on SNP markers derived from the transcribed portion of the genome. In other words, these SNP markers represented genic regions which occupy only a small portion, compared to the repetitive DNA which represent >80% of the wheat genome. The genetic gaps between linkage groups may represent genomic regions with a significant amount of repeat elements. Similar observations have been made in other studies using the same wheat Illumina iSelect 90K SNP assay (Kumar *et al.* 2016; Wen *et al.* 2017; Liu *et al.* 2018). Second, the RIL population used in this study was derived from a cross between two elite cultivars which were developed for the Midwest region of the United States, meaning there is only limited genetic variation between them and thus low level of polymorphism markers.

### Genetics of Grain Protein Content

Improving grain protein content is one of the principal objectives of most wheat breeding programs. Previous studies have reported few major and several minor QTL for grain protein content, suggesting the polygenic nature and quantitative inheritance of this trait (Johnson *et al.* 1978; Bogard *et al.* 2013; Echeverry-Solarte *et al.* 2015; Li *et al.* 2016). The most significant A-QTL in this study, *AQ.GPC.ndsu.5B*, identified on chromosome 5B, was also involved in a digenic epistatic interaction. Previous studies have reported an A-QTL associated with grain protein content on the long arm of chromosome 5B (Kulwal *et al.* 2005; Bordes *et al.* 2013; Echeverry-Solarte *et al.* 2015). However, unlike previous studies, this study identified the *AQ.GPC.ndsu.5B* A-QTL on the short arm of chromosome 5B, suggesting the novelty of this major A-QTL. Similar to our results, Prasad *et al.* (2003) and Groos *et al.* (2003) reported an A-QTL for grain protein content on chromosome 7A (Table 5). It is worthwhile to note that the minor stable A-QTL, *AQ.GPC.ndsu.7A*, showed nucleotide sequence similarity with the wheat HMGB1 protein. Christov *et al.* (2007) reported the wheat HMGB1 protein may play a major role in controlling general aspects of gene expression through chromatin structure modification. In addition to this significant role, Christov *et al.* (2007) also mentioned this protein possibly has a specific function as a general regulator of gene expression during cold stresses. Further studies are needed to elucidate the similarity between the *AQ.GPC.ndsu.7A* A-QTL and the wheat HMGB1 protein. As it was expected, most of the alleles for increased grain protein content were contributed by the cultivar Glenn.

### Genetics of Flour Extraction Rate and Mixograph-related Parameters

Flour extraction rate and mixograph-related parameters are important end-use quality traits for the milling industries. Both flour extraction and mixograph-related parameters are quantitative traits controlled by multiple genes (Campbell *et al.* 2001; Breseghello *et al.* 2005; Breseghello and Sorrells 2006; Simons *et al.* 2012; Echeverry-Solarte *et al.* 2015). This study found one stable A-QTL (*AQ.FE.ndsu.3B*) on chromosome 3B for flour extraction. Similarly, Carter *et al.* (2012) and Ishikawa *et al.* (2014) also reported a stable A-QTL with a minor effect on chromosome 3B for flour extraction (Table 5). Besides the A-QTL, this study also identified a stable DE-QTL (*DEQ.FE.ndsu.5A1/1D1*) for flour extraction. In addition, the *AQ.BLV.ndsu.5A* A-QTL, which showed a significant main effect for bread loaf volume, was involved in the epistatic interaction of the *DEQ.FE.ndsu.5A1/1D1* DE-QTL. Xing *et al.* (2014) indicated epistatic interactions could play an important role in the genetic basis of complex traits. Xing *et al.* (2002) and Yu *et al.* (1997) also mentioned epistatic effects should be much more sensitive to environmental effects than to main effects, making the detection of a stable QTL with an epistatic effect more difficult. This study is likely the first to report that a stable QTL with an epistatic effect for flour extraction. The majority of the positive alleles for flour extraction were contributed from the Traverse cultivar.

Previous studies have shown the effects of HMW-GS and LMW-GS on mixograph-related parameters (Payne *et al.* 1981; Brett *et al.* 1993; Gupta and MacRitchie 1994; Ruiz and Carrillo 1995; Maucher *et al.* 2009; Zhang *et al.* 2009; Branlard *et al.* 2001; He *et al.* 2005; Liu *et al.* 2005; Mann *et al.* 2009; Jin *et al.* 2013; Echeverry-Solarte *et al.* 2015; Jin *et al.* 2016). In the current study, a stable A-QTL (*AQ.MMLPT.ndsu.1B*) with a major effect on mixograph MID line peak time was detected on chromosome 1B, close to the location of the Glu-B1 gene encoding for HMW-GS. Similarly, a recent study reported a major stable A-QTL for mixograph MID line peak time in the same region close to the Glu-B1 gene (Jin *et al.* 2016). The favorable alleles for this A-QTL were contributed through the Glenn cultivar. The three stable A-QTL (*AQ.MMLPT.ndsu.2D*, *AQ.MMLPT.ndsu.3B.2*, and *AQ.MMLPT.ndsu.5D*) for mixograph MID line peak time on chromosomes 2D, 3B, and 5D, respectively, seem to be novel, with Traverse contributing the desirable alleles. In addition to the A-QTL, this study identified two novel stable epistatic DE-QTL (*DEQ.MMLPT.ndsu.2A2/4B1* and *DEQ.MMLPT.ndsu.4A1/5A1*) for mixograph MID line peak time on pairs of linkage groups 2A2/4B1 and 4A1/5A1, respectively. In another study, El-Feki *et al.* (2015) identified a significant epistatic interaction between the Glu-B1 locus on chromosome B1 and a QTL region near the microsatellite marker *Xwmc76* on chromosome 7B for mixograph MID line peak time in a doubled haploid hard winter wheat population.

### Genetics of Baking Properties

Baking quality evaluations are the final assessments to allow breeders to determine the appropriateness of a new wheat line to be released and accepted by the end users. Despite the importance of baking quality, limited information is available on the genetic control of baking properties. Previous studies have indicated the effects of HMW-GS on baking properties (Campbell *et al.* 2001; Rousset *et al.* 2001; Huang *et al.* 2006; Mann *et al.* 2009; Tsilo *et al.* 2010). In the current study, the locations of two major A-QTL (*AQ.BMT.ndsu.1B* and *AQ.BMT.ndsu.1B.2*) for bake-mixing time were found to be close to the location of the Glu-B1 gene. Besides these two A-QTL, three stable A-QTL were detected for baking properties, *AQ.BA.ndsu.4D.1*, *AQ.BA.ndsu.1B*, and *AQ.BMT.ndsu.3A*. Similar to the *AQ.BMT.ndsu.1B* and *AQ.BMT.ndsu.1B.2* A-QTL for bake-mixing time, the favorable allele for the *AQ.BMT.ndsu.3A* A-QTL was contributed by Glenn cultivar. Conversely, the favorable alleles for the *AQ.BA.ndsu.4D.1* and *AQ.BA.ndsu.1B A-QTL* were contributed by Traverse cultivar. Similar results were reported by Kuchel *et al.* (2006) and Tsilo *et al.* (2011) who found A-QTL for baking absorption on chromosome 1B (Table 5). The previous studies reported A-QTL for bread loaf volume on every wheat chromosome except chromosomes 3D, 4A, 5A, and 6A (Mann *et al.* 2009; Simons *et al.* 2012; Tsilo *et al.* (2011)). Unlike these reports, our study found a major A-QTL (*AQ.BLV.ndsu.5A*) for bread loaf volume on chromosome 5A. This study found one A-QTL with minor effect (*AQ.CBCL.ndsu.6B*) on chromosome 6B for crumb color. This A-QTL was located very close to the position of the A-QTL (*gwm193*) that Groos *et al.* (2007) reported for crumb grain score. In the current study, for the first time, a stable A-QTL (*AQ.BMT.ndsu.5D*) was identified on chromosome 5D for bake-mixing time. Two novel major A-QTL (*AQ.CTCL.ndsu.6B.1* and *AQ.CTCL.ndsu.7A*) on chromosomes 6B and 7A were detected for crust color. To our knowledge, there is no previous works reporting the digenic epistatic interaction effects for baking properties. Our study showed a total of 15 DE-QTL were identified addressing this issue confirming the complex nature of inheritance of the baking properties of wheat flour.

### Closely Linked or Pleiotropic Effects

Pleiotropic QTL could be valuable in the simultaneous improvement of several traits. Our results showed most of the end-use quality traits were associated with each other. Thus, it was expected to be able to identify co-localized (closely linked or pleiotropic) QTL controlling these traits. A total of 19 additive co-localized QTL were identified for the end-use quality traits in the current study. This is results is in agreement with previous studies (Cheverud 2000; Leamy *et al.* 2002; Wolf *et al.* 2006) who reported that most of these additive co-localized QTL (∼74%) showed positive pleiotropy. The loci controlling functionally integrated groups of traits are known to show positive pleiotropy (Cheverud 2000; Leamy *et al.* 2002; Wolf *et al.* 2006). However, five additive pleiotropic loci showed negative pleiotropy in the current study. These five additive co-localized QTL harbored A-QTL for grain protein content and flour extracion; grain protein content and bake-mixing time; MMPLT and grain protein content; flour extraction, baking absorption, and mixograph MID line time * value; and baking absorption, mixograph envelope right slope, and mixograph envelope left slope on chromosomes 1A, 1B, 2A, 4A, and 4D, respectively. Similar results were reported by Echeverry-Solarte *et al.* (2015) who found a co-localized QTL with negative pleiotropy on chromosome 5B for three integrated sets of traits (grain protein content, mixograph envelope peak time, and mixograph MID line peak time, where alleles from the exotic parent (WCB617) increased grain protein content, but decreased mixograph envelope peak time and mixograph MID line peak time. In the current study, the most important co-localized QTL was identified on chromosome 1B, which harbored two major A-QTL (*AQ.BMT.ndsu.1B.2* and *AQ.MMLPT.ndsu.1B*) for bake-mixing time and mixograph MID line peak time, respectively. Moreover, this co-localized QTL was located very close to the location of the Glu-B1 gene. Furthermore, this showed positive pleiotropy, where the desirable alleles were contributed through the Glenn cultivar. This positive pleiotropy indicated that a simultaneous improvement of bake-mixing time and MMPLT would be possible through selection. Besides the additive co-localized QTL, four epistatic co-localized QTL were identified in the current study. It is generally accepted that additive pleiotropic effects are more common than epistatic pleiotropic effects (Wolf *et al.* 2005 and 2006). Thus, as expected, the frequency of epistatic co-localized QTL was less than the frequency of additive co-localized QTL. The current study appears to be the first to report for epistatic co-localized QTL for end-use quality traits in wheat. Furthermore, all epistatic showed positive pleiotropy effect except one, which harbored A-QTL on pairs of linkage group 1A1/7D3 for grain protein content and mixograph envelope right slope. This negative pleiotropy is in contrast with previous findings; Wolf *et al.* (2005) suggested positive pleiotropy might be generally expected in epistatic pleiotropic analyses of integrated sets of traits.

### Conclusion

The current study suggests that flour extraction, mixograph envelope right slope, mixograph MID line peak time, and bake-mixing time can be used for the evaluation of the end-use quality traits in wheat breeding programs due to their high broad-sense heritability values. Overall, both parental lines (Glenn and Traverse) contributed desirable alleles that had positive effects on the end-use quality traits, suggesting both parental lines could be excellent resources to improve end-use quality traits in wheat breeding programs.

In the current study, a much improved high-density SNP-based linkage map was constructed and used to identify QTL for end-use quality traits in wheat. It is worthwhile to note the use of the wheat Illumina 90K iSelect assay resulted in a better improvement in genome coverage, marker density, and identification of QTL compared to previous studies for end-use quality traits in wheat.

This study identified 12 stable major main effect QTL and three stable digenic epistatic interactions for the end-use quality traits in wheat. This suggests that both additive and digenic epistatic effects should be considered for these traits in molecular wheat breeding programs, such as MAS. Furthermore, a total of 23 closely-linked or pleiotropic loci were identified in this study. The co-localized QTL could be valuable to simultaneously improve the end-use quality traits via selection procedures in wheat breeding programs. The information provided in the current study could be used in molecular wheat breeding programs to enhance selection efficiency and to improve the end-use quality traits in wheat.
